# Spermidine Targets Ovarian Granulosa Cells via Activating the FHC/SLC7A11 Axis to Regulate Iron Homeostasis and Ameliorate Iron Overload-Induced Ovarian Dysfunction

**DOI:** 10.3390/antiox15050637

**Published:** 2026-05-18

**Authors:** Chun-Yang Niu, Dong-Mei Jiang, Xin Wang, Guan-Hua Chen, Shuo Li, Yong-Ni Guo, Cheng-Weng Ji, Xiao-Guang An, Wei-Kang Ling, Yu-Xin Qi, Xin-Yi Wang, Lu Lu, Xun Wang, Bo Kang

**Affiliations:** 1 State Key Laboratory of Swine and Poultry Breeding Industry, College of Animal Science and Technology, Sichuan Agricultural University, Chengdu 611130, China; 2020102008@stu.sicau.edu.cn (C.-Y.N.); xinwang@stu.sicau.edu.cn (X.W.); 2023202044@stu.sicau.edu.cn (S.L.); 2020202046@stu.sicau.edu.cn (Y.-N.G.); jichengweng@stu.sicau.edu.cn (C.-W.J.); anxiaoguang@stu.sicau.edu.cn (X.-G.A.); lingweikang@stu.sicau.edu.cn (W.-K.L.); qiyuxin@stu.sicau.edu.cn (Y.-X.Q.); wangxinyi7@stu.sicau.edu.cn (X.-Y.W.); lu.lu@sicau.edu.cn (L.L.); xunwang@sicau.edu.cn (X.W.); 2 Department of Animal Science, College of Smart Animal Husbandry, College of Biology and Food, Shangqiu Normal University, Shangqiu 476000, China; chenguanhua2014@126.com; 3 Animal Nutrition Institute, Sichuan Agricultural University, Chengdu 611130, China

**Keywords:** ovary, ferroptosis, spermidine, granulosa cells, SLC7A11

## Abstract

Females with iron overload suffer from follicular dysplasia, and effective therapeutic strategies for preserving fertility remain lacking. As a natural aliphatic polyamine, spermidine exerts antioxidant activity and plays an anti-ferroptosis role in the pathogenesis of various diseases. However, the role and underlying mechanism of spermidine in iron overload-induced ovarian ferroptosis remain largely elusive. This study aimed to investigate the therapeutic potential of spermidine against iron overload-induced ferroptosis in ovarian granulosa cells and elucidate its molecular mechanism. As a result, iron overload models were established in female mice (in vivo, ferrous sulfate) and porcine ovarian granulosa cells (in vitro, ferric ammonium citrate), with spermidine administered at 3 mM (in vivo) or 150 μM (in vitro). Ferritin heavy chain (FHC) and solute carrier family 7 member 11 (SLC7A11) silencing were performed via siRNA transfection, and relevant controls were set. In vivo studies showed that spermidine elevated serum estradiol and progesterone levels, enhanced ovarian catalase (CAT) and superoxide dismutase (SOD) activities, improved granulosa cell mitochondrial morphology, and increased estrous cycle regularity from 35.6% (high-iron group) to 63.1%. In vitro, spermidine improved ferric ammonium citrate (FAC)-impaired cell viability; attenuated reactive oxygen species (ROS) accumulation; upregulated FHC, Nrf2/p-Nrf2/GPX4, SLC7A11 and anti-müllerian hormone (AMH) expression; and inhibited excessive autophagy (decreased LC3BII/I ratio). Mechanistically, spermidine activated AKT-mediated autophagy, modulated iron homeostasis and glutathione (GSH) synthesis via FHC, alleviated ferroptosis-related Nrf2/p-Nrf2/HO-1 pathway overactivation, reduced lipid peroxidation and DNA damage, and restored mitochondrial function. SLC7A11 silencing disrupted glutathione metabolism, induced mitochondrial ROS accumulation, and inhibited autophagy. Proteomic analysis identified microsomal glutathione S-transferase 3 (MGST3) as a potential key downstream target of spermidine in suppressing SLC7A11-mediated ferroptosis. This study reveals a novel therapeutic strategy wherein spermidine protects against ovarian ferroptosis and preserves ovarian function by regulating iron homeostasis through the FHC/SLC7A11 axis.

## 1. Introduction

Infertility has become a global public health challenge. Ferroptosis is a regulated form of cell death triggered by iron overload and excessive reactive oxygen species (ROS). Its progression is tightly modulated by iron homeostasis, the cystine/glutamate antiporter (Xc^−^ system) [1], autophagy, and other signaling pathways [2,3,4]. Accumulating clinical evidence has demonstrated that excessive iron deposition in serum and ovarian tissues acts as a critical pathogenic factor for female infertility. Iron overload is estimated to account for 5–15% of unexplained female infertility cases in the general population, especially in patients with endometriosis [5,6]. In high-risk populations, including individuals with hereditary hemochromatosis and transfusion-dependent thalassemia, 30–50% of clinical infertility cases can be explicitly attributed to systemic and ovarian iron overload [7]. Excessive iron not only impairs the endocrine system but also leads to hypogonadotropic hypogonadism in females [8].

Ferroptosis is characterized by elevated intracellular iron levels, ROS overload, and excessive lipid peroxidation, ultimately leading to cell death [9]. Iron in the labile iron pool (LIP) can be stored in ferritin, utilized in mitochondria, or exported out of cells via ferroportin (FPN) [10]. Oocyte-specific deficiency of basic nucleoprotein 1 (BNC1) upregulates acyl-coa synthetase long-chain family member 4 (ACSL4) and transferrin receptor (TfR1) expression through the NF2/YAP pathway. This process induces iron overload and lipid peroxidation in oocytes, thereby triggering ferroptosis [11]. Ferroptosis-related genes are also involved in the regulation of granulosa cell proliferation and ovarian reserve function.

Ferritin heavy chain (FHC, also termed FTH) is a core ferritin subunit with ferrous oxidase activity. It maintains cellular iron homeostasis and plays a critical regulatory role in ferroptosis. The iron transporter solute carrier family 40, member 1 (SLC40A1) and the iron storage proteins FHC/FTL (ferritin light chain) are transcriptionally regulated by nuclear factor erythroid 2-related factor 2 (Nrf2) [12]. Nrf2 knockdown reduces the expression of iron transporters and FTL/FHC, inhibits iron storage and excretion, increases free iron levels, and promotes the Fenton reaction and subsequent ROS production [13,14]. The solute carrier family 7 member 11 (SLC7A11, xCT) and solute carrier family 3 member 2 (SLC3A2) form the cellular antioxidant Xc^−^ system, in which SLC7A11 serves as the core functional unit [15]. Cystine is transported into cells via SLC7A11 on the cell membrane, thereby promoting glutathione (GSH) synthesis [16]. Glutathione peroxidase 4 (GPX4) uses GSH to convert toxic lipid peroxides (L-OOH) into nontoxic alcohols (L-OH). This effect eliminates peroxide stress and suppresses ferroptosis [17]. Studies have demonstrated that Nrf2 upregulates membrane-localized SLC7A11, reduces lipid peroxidation products, promotes cystine transport, increases GSH) levels, and upregulates GPX4, thus exerting anti-ferroptotic effects [18,19]. Iron overload has been detected in follicular fluid from stage III/IV endometriosis patients with infertility. Treatment of human granulosa cells with such follicular fluid markedly inhibits cell proliferation and migration [6]. These findings indicate that iron overload in follicular fluid is a key driver of granulosa cell dysfunction and endometriosis-associated infertility.

Spermidine is a low-molecular-weight aliphatic nitrogen-containing compound widely distributed in animals and is well recognized for its antioxidant properties. Spermidine binds to and stabilizes negatively charged molecules (e.g., RNA, DNA, ATP), thereby regulating a variety of physiological and pathological processes [20]. A study published in *Science* reported that dietary intake of exogenous spermidine acts as an effective ROS scavenger [21]. Intracellular spermidine is strictly controlled at physiological levels. Owing to its anti-inflammatory, antioxidant and mitochondrial protective properties, spermidine regulates transcription, translation, oxidative stress, autophagy, apoptosis and genomic stability [22,23]. In female animals, decreased ovarian polyamine levels lead to follicular development arrest. However, the underlying mechanism by which spermidine modulates iron overload-induced ferroptosis in ovarian granulosa cells remains largely unknown.

Ferric ammonium citrate (FAC) mimics non-transferrin-bound iron to increase intracellular iron levels, leading to ferroptosis and impaired cell differentiation. Excessive ferrous iron induces the production of ROS and lipid peroxidation (LPO) products, which cause typical mitochondrial morphological changes associated with ferroptosis, such as membrane shrinkage, cristae reduction or loss, and outer membrane rupture [24]. Mice were selected for in vivo ovarian functional validation due to their conserved iron metabolic and ovarian physiological pathways, well-defined genetic background, easy manipulation and wide application in reproductive ferroptosis research. Considering the inherent species differences between rodent and human ovarian granulosa cells in iron homeostasis regulation and endocrine characteristics, primary porcine ovarian granulosa cells were adopted for in vitro mechanistic exploration, as pigs exhibit high similarity to humans in follicle physiology, iron metabolism and cellular ferroptosis regulation, which improves the translational reliability of the present findings. Therefore, in this study, we established iron overload models in mouse ovaries and porcine ovarian granulosa cells to investigate the protective effect of spermidine against iron overload-induced ovarian damage and elucidate its underlying molecular mechanism.

## 2. Materials and Methods

### 2.1. Reagents

Spermidine (S0266), ferric ammonium citrate (FAC), chloroquine (CQ, C6628), anti-LC3B (L7543), Rapamycin (Rapa), trans-4-methylcyclohexylamine (Trans) were obtained from Sigma (St. Louis, MO, USA). Ferrostatin-1 (Fer-1) were acquired from MedChemExpress (South Brunswick, NJ, USA). Perifosine (AKT inhibitor) was obtained from Solarbio (Beijing, China). Mito-FerroGreen fluorescence probe and FerroOrange were obtained from Dojindo (Kumamoto, Japan). Lipid Peroxidation (LPO) assay kit was from Nanjing Jiancheng Bioengineering Institute (Nanjing, China). MitoSOX Red Mitochondrial Superoxide Indicator was bought from Yeasen (Shanghai, China). Cell counting kit 8 (CCK-8), Mito-Tracker Red CMXRos, GSH and glutathione disulfide (GSSG) assay kit, Bicinchoninic acid (BCA) protein assay kit, 4′,6-diamidino-2-phenylindole (DAPI) staining solution, 5,5′,6,6′-Tetrachloro-1,1′,3,3′-tetraethylbenzimidazolylcarbocyanine iodide (JC-1) mitochondrial transmembrane potential assay kit and Hoechst 33342 were purchased from Beyotime Biotechnology (Shanghai, China). The primary antibodies used for Western blotting and immunofluorescence experiments are listed in Table 1.

### 2.2. Animals and Experimental Design

Seven-week-old female specific pathogen-free ICR mice (*n* = 125, body weight 21.0 ± 0.5 g) were purchased from Ensville Biotechnology Co., Ltd. (Chengdu, China). All mice were housed in the animal facility of the Animal Management Center with free access to food and water. The housing conditions were maintained at 23 ± 2 °C with a relative humidity of 65 ± 5% and a 12 h light/dark cycle. After one week of adaptive feeding, 125 female mice were randomly divided into 5 groups (*n* = 25/group): control group (0.045 g/kg ferrous sulfate of diet), medium-iron group (MFe, 0.55 g/kg ferrous sulfate of diet), high-iron group (HFe, 1.350 g/kg ferrous sulfate of diet), high-iron + 3 mM spermidine group (3SH), and high-iron + 6 mM spermidine group (6SH). Mice were fed with diets containing different concentrations of ferrous sulfate, and spermidine was dissolved in sterile water for oral administration; the control group received an equal volume of sterile water. The treatment lasted for 8 weeks.

During the experiment, the general condition of the mice was observed daily; body weight was recorded every 4 days, and food intake was monitored regularly. From the 7th week onwards, vaginal smears were collected for 3 consecutive weeks to observe the estrous cycle (10 mice per group were used for estrous cycle monitoring). At the end of the treatment period, mice were anesthetized by intraperitoneal injection of pentobarbital sodium, and blood was collected via the orbital venous plexus. Serum was separated by centrifugation and stored at −80 °C for subsequent analysis. Ovaries and surrounding adipose tissues were isolated under a stereomicroscope (Olympus, Tokyo, Japan); bilateral ovaries were completely separated, weighed, and photographed using a digital microscope (Motic, Xiamen, China). The left ovary was fixed for paraffin sectioning, and the right ovary was frozen in liquid nitrogen and stored at −80 °C for subsequent molecular and biochemical assays.

### 2.3. Monitoring of the Estrous Cycle in Mice

From the 7th week of treatment, vaginal smears were collected every morning for 3 consecutive weeks to monitor the estrous cycle (10 mice per group). Vaginal smears were prepared and observed under a light microscope (Nikon, Tokyo, Japan) according to a previously described method [25]. The normal estrous cycle of female Institute of Cancer Research (ICR) mice is 4 ~ 5 days, including proestrus, estrus, metestrus, and diestrus.

### 2.4. Detection of Iron Content in Mouse Ovary and Serum

Serum and ovarian iron contents were measured using commercial assay kits according to the manufacturer’s instructions, including the serum iron assay kit (A039-1-1, Nanjing Jiancheng Bioengineering Institute, Nanjing, China) and ovarian tissue iron assay kit (A039-2-1, Nanjing Jiancheng). All assays were performed in triplicate.

### 2.5. Histomorphological Observation and Follicle Counting

Ovarian tissues were fixed in 4% paraformaldehyde for 24 h, embedded in paraffin, and sectioned into 5 μm slices. Hematoxylin and eosin (HE) staining was performed according to a previously described method, and images were captured using a light microscope [25]. Follicle counting was conducted by two independent investigators in a blinded manner; follicles were classified as primordial follicles, primary follicles, secondary follicles, atretic follicles, and corpus luteum according to morphological criteria.

### 2.6. Measurement of Hormone Levels

Mouse serum estradiol (E2) and progesterone (P4) levels were measured using mouse E2 ELISA kit (Mlbio, ml001962, Shanghai, China) and mouse P4 ELISA kit (Mlbio, ml057778, Shanghai, China), respectively, according to the manufacturer’s instructions. Porcine granulosa cell lysates and culture supernatants were collected to detect E2 and P4 levels using porcine E2 ELISA kit (ml002366, Shanghai Enzyme-linked Biotechnology Co., Ltd., Shanghai, China) and porcine P4 ELISA kit (ml002422, Shanghai Enzyme-linked Biotechnology), respectively. The inter-assay and intra-assay coefficients of variation were <15% and <10%, respectively. The detection sensitivities of the P4 and E2 ELISA kits were 0.1 ng/mL and 0.1 pM, respectively. All assays were performed in triplicate.

### 2.7. Culture and Treatment of Porcine Ovarian Granulosa Cells

Porcine ovarian granulosa cells were isolated from healthy porcine ovaries (collected from a local abattoir) and cultured in F12/DMEM medium supplemented with 10% fetal bovine serum (FBS) and 1% penicillin–streptomycin in a humidified incubator at 37 °C with 5% CO_2_. When porcine ovarian granulosa cells reached 70% confluence, the cells were treated with gradient concentrations of FAC (0, 50, 100, 200, 400, 800 μM) for 24 h or 36 h to screen the optimal concentration and duration for ferroptosis induction. For spermidine intervention, cells were cultured in serum-free medium and treated with different concentrations of spermidine (50, 100, 150, 200 μM) combined with 200 μM FAC for 36 h. For functional experiments: cells were treated with 1 μM Fer-1 (a ferroptosis inhibitor) for 36 h; co-treated with 150 μM spermidine and 10 μM chloroquine (CQ) (an autophagy inhibitor) for 36 h; treated with 200 μM FAC combined with 10 μM Rapa (an autophagy inducer) for 36 h; or pre-treated with 1 μM Trans (a spermidine synthase inhibitor) for 24 h followed by subsequent treatments.

### 2.8. Measurement of Cell Viability

Cell viability was detected using the CCK-8 kit (Meilun Biotechnology, Dalian, China) according to the manufacturer’s instructions. Porcine granulosa cells were seeded in 96-well plates at a density of 4 × 10^3^ cells/well and treated as described above. After treatment, 10 μL of CCK-8 reagent was added to each well, and the plates were incubated at 37 °C for 2 h. The absorbance at 450 nm was measured using a microplate reader(Thermo Fisher Scientific, Waltham, USA). Cell viability was calculated using the following formula: Cell viability (%) = (OD_450_ of experimental group − OD_450_ of blank group)/(OD_450_ of control group − OD_450_ of blank group) × 100%, where the blank group contained only medium without cells. All experiments were performed in triplicate with three independent replicates.

### 2.9. Determination of Ovarian Antioxidant Capacity

Mouse ovarian tissues (two ovaries per sample) were weighed and homogenized in cold physiological saline, then centrifuged at 2500 rpm for 10 min at 4 °C to collect the supernatant. The activities of malondialdehyde (MDA), superoxide dismutase (SOD), and catalase (CAT) in the ovarian homogenate supernatant were measured using commercial assay kits (Nanjing Jiancheng Bioengineering Institute, Nanjing, China) according to the manufacturer’s instructions. All assays were performed in triplicate.

### 2.10. Intracellular Reactive Oxygen Species Detection

Intracellular ROS levels in porcine granulosa cells were detected using the ROS assay kit (Beyotime Biotechnology) according to the manufacturer’s protocol. Briefly, cells were rinsed three times with pre-cooled phosphate-buffered saline (PBS) to remove drug residues, then incubated with 10 μM DCFH-DA working solution at 37 °C for 30 min in the dark. Rosup (50 mg/mL, serum-free medium) was added to untreated cells as a positive control. After incubation, cells were washed three times with F12 medium, and ROS fluorescence (green) was observed and photographed using an Olympus digital section microscope (VS120-S6-W, Olympus, Tokyo, Japan). ROS fluorescence intensity was quantified using the ImageJ software (version 1.54f, National Institutes of Health, Bethesda, MD, USA). All experiments were performed in triplicate.

### 2.11. Measurement of Fe^2+^ Level in Granulosa Cells

Intracellular Fe^2+^ levels were detected using the FerroOrange fluorescent probe (F374, Dojindo), a specific live-cell Fe^2+^ imaging probe. Porcine granulosa cells were cultured overnight in a 24-well plate at 37 °C with 5% CO_2_, then washed three times with F12 medium. Cells were treated with the indicated drugs, then incubated with 1 μM FerroOrange working solution at 37 °C for 30 min in the dark without washing. Fe^2+^ fluorescence (red) was immediately observed and imaged under a fluorescence microscope(Model IX73-U, Olympus, Tokyo, Japan). Fluorescence intensity was quantified using the ImageJ software. All experiments were performed in triplicate.

### 2.12. Determination of Lipid Peroxidation Level in Granulosa Cells

Intracellular lipid peroxidation levels were measured using the LPO assay kit (Nanjing Jiancheng, A106-1-2) according to the manufacturer’s instructions. Briefly, porcine granulosa cells were collected by trypsin digestion, resuspended in 0.8 mL PBS, and centrifuged at 1000 r/min for 10 min; the cell pellet was retained and washed twice with PBS. A portion of the cell pellet was lysed with radio immunoprecipitation assay (RIPA) lysis buffer for 40 min, and the protein concentration was measured using the BCA assay kit. Another portion of the cell pellet was lysed with Triton X-100 lysis buffer, and LPO levels were detected according to the kit instructions. All assays were performed in triplicate.

### 2.13. Transmission Electron Microscopy

Porcine granulosa cells were collected after treatment and fixed in 3% glutaraldehyde at 4 °C for 24 h, then post-fixed in 1% osmium tetroxide. The cells were dehydrated with a graded ethanol series, embedded in epoxy resin, and sectioned into ultrathin slices (70 nm). The sections were stained with uranyl acetate and lead citrate, then observed and photographed under a transmission electron microscope (JEOL Ltd., Tokyo, Japan). Mitochondrial morphology was analyzed by two independent investigators in a blinded manner.

### 2.14. Detection of Mitochondrial Membrane Potential

Mitochondrial membrane potential (MMP) was detected using the JC-1 mitochondrial transmembrane potential assay kit (Beyotime, C2003S) according to the manufacturer’s instructions. Porcine granulosa cells were seeded in laser confocal dishes and treated as indicated. A positive control group (treated with carbonyl cyanide m-chlorophenylhydrazone, CCCP) was set up. After treatment, cells were incubated with JC-1 working solution at 37 °C for 20 min in the dark, then washed three times with JC-1 staining buffer. Fluorescence images (red for aggregated JC-1, green for monomeric JC-1) were captured using a laser confocal microscope (Carl Zeiss, Oberkochen, Germany), and the red/green fluorescence intensity ratio was calculated to represent MMP levels. All experiments were performed in triplicate.

### 2.15. Detection of Mitochondrial Fe^2+^ Levels

Mitochondrial Fe^2+^ levels were detected using the Mito-FerroGreen fluorescent probe (M489, Dojindo), a specific probe for mitochondrial Fe^2+^. Briefly, 53 μL of DMSO (dimethyl sulfoxide) was added to 50 μg of Mito-FerroGreen powder to prepare a 1 mM stock solution, which was diluted with serum-free medium to a 5 μM working solution. Porcine granulosa cells were incubated with 5 μM Mito-FerroGreen working solution at 37 °C for 30 min, then treated with FAC and/or spermidine. Mitochondrial Fe^2+^ fluorescence (green) was observed and imaged under a fluorescence microscope, and fluorescence intensity was quantified using the ImageJ software. All experiments were performed in triplicate.

### 2.16. Small Interfering RNA Transfected Granulosa Cells

Porcine granulosa cells were seeded in 12-well plates and cultured to 50% confluence in serum-free medium for siRNA transfection. For each well, 1.5 μL of siRNA (FHC siRNA, SLC7A11 siRNA, or negative control siRNA [siNC]) was mixed with 50 μL of Opti-MEM medium, and 1.5 μL of Lipofectamine 3000 was mixed with 50 μL of Opti-MEM medium. The two mixtures were combined and incubated at room temperature for 15 min, then added to the 12-well plate (100 μL/well). The cells were incubated at 37 °C with 5% CO_2_ for 48~72 h, and gene and protein expression levels were detected to verify transfection efficiency.

### 2.17. Detection of Reduced Glutathione/Oxidized Glutathione (GSH/GSSG) in Granulosa Cells

Intracellular reduced GSH and oxidized GSSG levels were measured using the GSH/GSSG assay kit (Beyotime, S0053) according to the manufacturer’s instructions. Porcine granulosa cells were collected and lysed with the kit’s lysis buffer, and the supernatant was collected by centrifugation. GSH and GSSG levels were detected by colorimetry, and the GSH/GSSG ratio was calculated to reflect intracellular redox status. All assays were performed in triplicate.

### 2.18. Determination of Mitochondrial Morphology and Mitochondrial Superoxide in Granulosa Cells

Mitochondrial morphology was detected using the Mito-Tracker Red CMXRos fluorescent probe (C1049B, Beyotime Biotechnology, Shanghai, China). Porcine granulosa cells were seeded in 24-well plates and cultured to 50% confluence, then incubated with Mito-Tracker Red CMXRos working solution at 37 °C for 20 min. The working solution was removed, preheated cell culture medium was added, and mitochondrial morphology was observed and photographed under a fluorescence microscope. Mitochondrial morphology was classified as Cat.I (normal slender reticular mitochondria), Cat.II (mild fragmentation), or Cat.III (severe fragmentation with perinuclear mitochondrial debris).

Mitochondrial superoxide levels were detected using the MitoSOX Red Mitochondrial Superoxide Probe (Yeasen, 40778ES50) according to the manufacturer’s instructions. Briefly, cells were incubated with 1 mL of MitoSOX Red working solution at 37 °C for 10 min in the dark, then washed three times with preheated F12 medium. Cells were counterstained with 10 μL of DAPI staining solution for 15 min, mounted, and observed under a fluorescence microscope. Mitochondrial superoxide fluorescence (red) intensity was quantified using the ImageJ software. All experiments were performed in triplicate.

### 2.19. Western Blotting

Mouse ovarian tissues or porcine granulosa cells were collected and lysed with RIPA lysis buffer containing 1% protease and phosphatase inhibitor cocktail (Beyotime Biotechnology) on ice for 30 min. The lysates were centrifuged at 12,000 rpm for 15 min at 4 °C, and the supernatant was collected. Protein concentration was measured using the BCA assay kit. Equal amounts of protein (30 μg/lane) were separated by SDS-PAGE (sodium dodecyl sulfate-polyacrylamide gel electrophoresis) and transferred to PVDF (polyvinylidene difluoride) membranes. The membranes were blocked with 5% non-fat milk in TBST (tris-buffered saline with Tween-20) for 1 h at room temperature, then incubated with the indicated primary antibodies at 4 °C overnight. After washing three times with TBST, the membranes were incubated with horseradish peroxidase (HRP)-conjugated secondary antibodies for 1 h at room temperature. Protein bands were visualized using an ECL chemiluminescence kit (Beyotime Biotechnology), and band intensity was quantified using the ImageJ software. β-Actin or β-tubulin was used as the internal reference. All experiments were performed in triplicate.

### 2.20. Cell Immunofluorescence

Porcine granulosa cells were seeded in 24-well plates with glass coverslips and treated with spermidine and/or FAC as indicated. After treatment, cells were washed three times with PBS, fixed with 400 μL of 4% paraformaldehyde for 10 min, and permeabilized with 0.1% Triton X-100 for 15 min. Cells were blocked with 5% bovine serum albumin (BSA) for 1 h at room temperature, then incubated with the indicated primary antibodies at 4 °C overnight. After washing three times with PBS (10 min each), cells were incubated with fluorescent secondary antibodies for 1 h in the dark at room temperature, then washed three times with PBS (10 min each). Nuclei were stained with DAPI for 10 min, and the coverslips were mounted on glass slides. Fluorescence images were captured using a fluorescence microscope, and fluorescence intensity was quantified using the ImageJ software. All experiments were performed in triplicate.

### 2.21. Proteome Sequencing

Porcine granulosa cells were divided into three groups (3 biological replicates per group): FAC group (siNC + 200 μM FAC), FAC + SPD group (siNC + 200 μM FAC + 150 μM spermidine), and siSLC7A11 + FAC + SPD group (siSLC7A11 + 200 μM FAC + 150 μM spermidine). After treatment, cells were collected and sent to Shanghai Baipu Biotechnology Co., Ltd., Shanghai, China, for proteomic sequencing. Briefly, the experimental steps included: (1) Protein preparation and TMT (tandem mass tag) labeling: cells were lysed on ice, homogenized, boiled, ultrasonicated, and centrifuged at 12,000 r/min for 15 min. Protein concentration was measured by the BCA assay, and proteins were digested with trypsin at 37 °C overnight. The resulting peptides were labeled with TMT tags. (2) High pH reversed-phase separation and LC-MS (liquid chromatography-mass spectrometry) analysis: TMT-labeled peptides were separated by Agilent 1290 HPLC (high performance liquid chromatography) with a C18 column using a linear gradient, then analyzed by LC-MS/MS. (3) Data analysis: LC-MS/MS raw data were analyzed using the Proteome Discoverer 2.4 software with a false discovery rate (FDR) of 1%. Differentially expressed proteins (DEPs) were screened with the criteria of fold change >1.20 or <0.83 and *p* < 0.05. Kyoto encyclopedia of genes and genomes (KEGG) pathway enrichment analysis was performed to identify the main signaling pathways associated with DEPs.

### 2.22. Statistical Analysis

All data were analyzed using the GraphPad Prism 7.0 software (San Diego, CA, USA). Data are presented as the mean ± standard deviation (SD). Differences between two groups were compared using Student’s *t*-test, and differences among multiple groups were compared using one-way analysis of variance (ANOVA) followed by Dunnett’s post hoc test. Prior to conducting ANOVA, normality of data distribution was assessed using the Shapiro–Wilk test, and homogeneity of variance was verified via Levene’s test. All datasets satisfied the assumptions of normal distribution and equal variance. *p* < 0.05 was considered statistically significant, and *p* < 0.01 was considered highly statistically significant. For multiple comparisons, the same letter indicates no significant difference (*p* > 0.05), and different letters indicate a significant difference (*p* < 0.05).

## 3. Results

### 3.1. Effects of Spermidine on Body Weight, Ovarian Index and Iron Content in Iron Overload Mice

**Figure 1:**
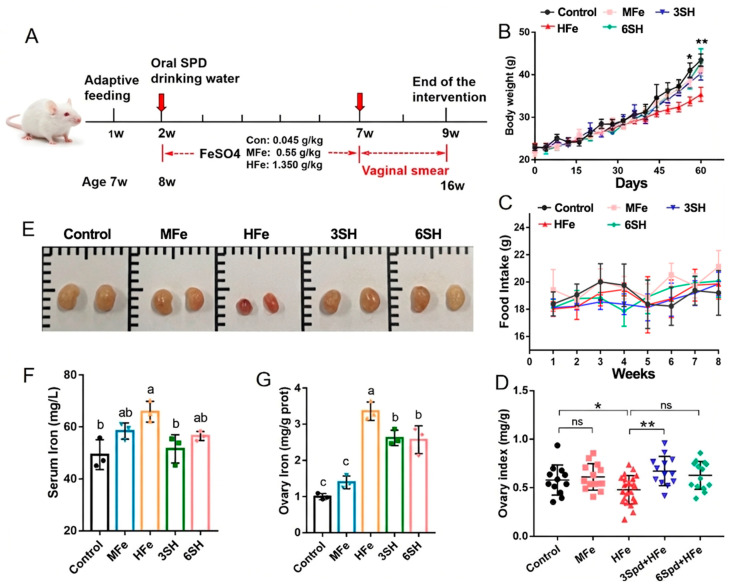
Effects of spermidine (SPD) on ovarian index and iron content in iron overload mice. (**A**) Schematic diagram of the animal model establishment. (**B**) Body weight of mice (*n* = 5). (**C**) Food intake of mice (*n* = 5). (**D**) Ovarian index of mice (*n* ≥ 12). (**E**) Representative images of mouse ovarian morphology. (**F**) Serum iron ion levels detected by flame atomic absorption spectrometry (*n* = 3). (**G**) Ovarian tissue iron ion levels detected by colorimetry (*n* = 3). Control group (0.045 g/kg ferrous sulfate), medium-iron group (MFe, 0.55 g/kg ferrous sulfate), high-iron group (HFe, 1.350 g/kg ferrous sulfate), high-iron + 3 mM spermidine group (3SH), and high-iron + 6 mM spermidine group (6SH). Data are presented as mean ± SD. * *p* < 0.05, ** *p* < 0.01; same letters indicate no significant difference (*p* > 0.05), different letters indicate significant difference (*p* < 0.05).

Trace element measurement confirmed that dietary iron content satisfied the experimental standard, with a marginal error less than 15% (Supplementary Table S1). The schematic diagram of the animal model establishment is shown in Figure 1A. Iron overload induced by 1.35 g/kg ferrous sulfate remarkably suppressed mice body weight gain relative to the control (*p* < 0.05). Conversely, 3 mM and 6 mM spermidine interventions effectively alleviated iron overload-mediated body weight reduction (*p* < 0.05; Figure 1B). No intergroup difference in food intake was detected across all treatments (*p* > 0.05; Figure 1C). The ovarian index was markedly increased in the 3SH group compared with the HFe group (*p* < 0.01), while the 6SH group showed no statistically significant alteration (*p* > 0.05; Figure 1D). Representative histological morphology of ovarian tissues is presented in Figure 1E. Furthermore, spermidine intervention substantially mitigated excessive iron deposition in serum and ovarian tissues under iron overload conditions. Both serum and ovarian iron levels were distinctly decreased in the 3SH group versus the HFe group (*p* < 0.05), and the 6SH group also achieved a significant reduction in ovarian iron concentration (*p* < 0.05; Figure 1F,G).

### 3.2. Effects of Spermidine on Ovarian Endocrine Function in Iron Overload Mice

**Figure 2:**
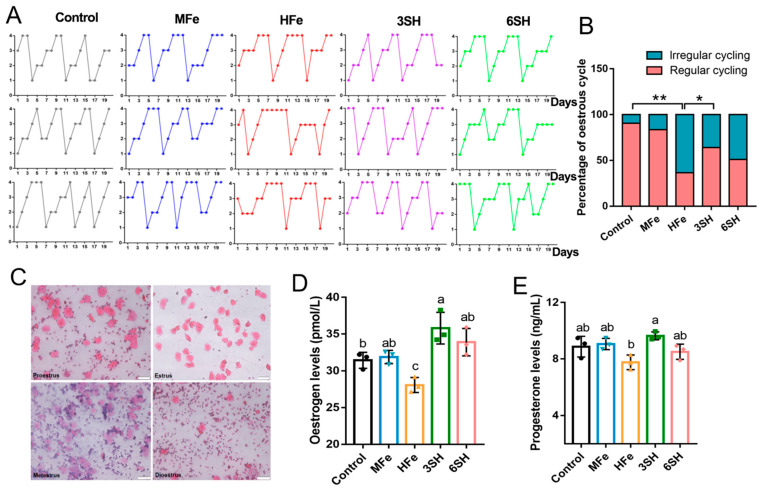
Effects of spermidine on ovarian endocrine function in iron overload mice. (**A**) Monitoring of mouse estrous cycle. The ordinate represents: 1. Proestrus; 2. Estrous period; 3. Metestrus; 4. Diestrus (*n* = 6). (**B**) Proportion of mice with regular estrous cycle (*n* = 10). (**C**) HE staining of mouse vaginal smears (Scale bar, 50 μm). (**D**) Serum estradiol levels and (**E**) serum progesterone levels in mice (*n* = 3). Data are presented as mean ± SD. * *p* < 0.05, ** *p* < 0.01, same letters indicate no significant difference (*p* > 0.05), different letters indicate significant difference (*p* < 0.05).

Ovarian endocrine function was assessed by monitoring sex hormone profiles and estrous cycle cyclicity. Estrous cycle stages were determined via hematoxylin–eosin (HE) staining of vaginal smears (Figure 2A,C). The control group exhibited a regular estrous cycle rate of 92.5%, whereas the HFe group displayed a markedly reduced cyclicity of only 35.6% (*p* < 0.05), which was significantly lower than that of the control and MFe groups. In comparison with the HFe group, the 3SH group presented a remarkably elevated proportion of regular estrous cycles (63.1%, *p* < 0.05), while the 6SH group also showed an upward trend in cyclicity recovery (Figure 2B). Serum hormonal analysis revealed suppressed estradiol accumulation in the HFe group relative to the control group (*p* < 0.05). Notably, spermidine intervention reversed iron-induced hormonal dysfunction: the 3SH group exhibited distinctly increased serum estradiol and progesterone levels (*p* < 0.05), and the 6SH group also obtained a significant elevation in serum estradiol concentration (*p* < 0.05; Figure 2D,E).

### 3.3. Effects of Spermidine on Histomorphology, Oxidative Stress and Ferroptosis-Related Proteins in Iron Overloaded Mice

**Figure 3:**
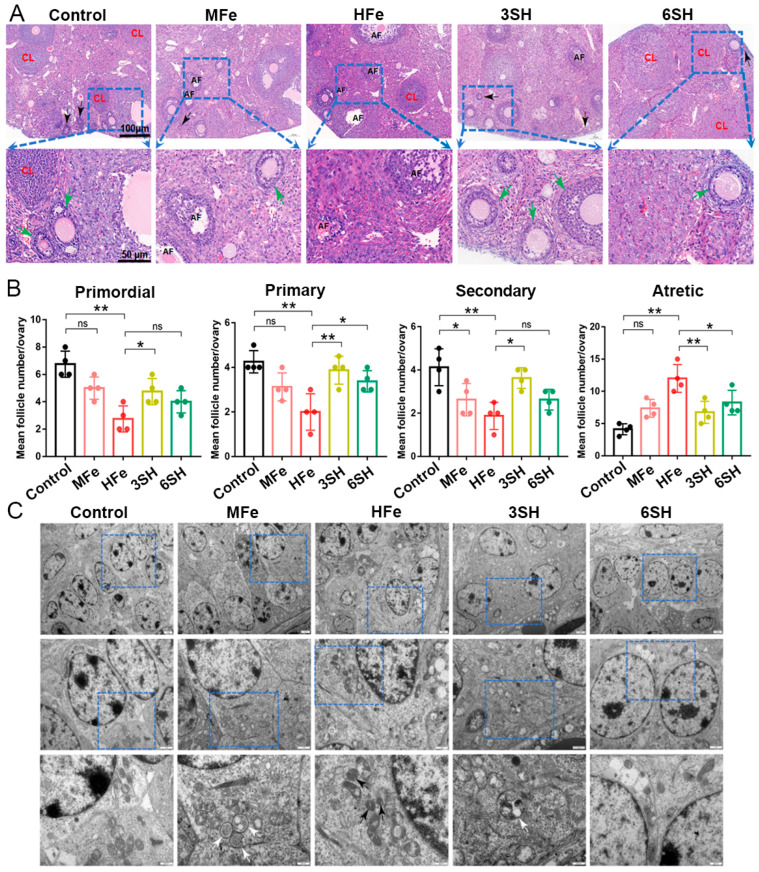
Effects of spermidine on ovarian follicle number and granulosa cell ultrastructure in mice. (**A**) Hematoxylin-eosin (HE) staining of ovarian tissue (CL, corpus luteum; AF, atretic follicles; green arrow, growing follicles; black arrow, primordial follicles; Scale bar, 100 μm [top] or 50 μm [bottom]). (**B**) Quantitative analysis of ovarian follicles at all stages (*n* = 4). (**C**) Transmission electron microscopy (TEM) observation of granulosa cell ultrastructure (white arrow, autophagosomes or autophagic lysosomes; black arrow, typical ferroptotic mitochondria; Scale bar, 2.0 μm [top], 1.0 μm [middle], 500 nm [bottom]; *n* = 3). Data are presented as mean ± SD. * *p* < 0.05, ** *p* < 0.01.

HE staining and follicle quantification demonstrated that iron overload induced severe ovarian follicular damage. Relative to the control group, the HFe group exhibited prominent depletion of primordial, primary, and secondary follicles as well as corpora lutea, accompanied by a marked accumulation of atretic follicles (*p* < 0.05; Figure 3A,B). In contrast, spermidine intervention effectively ameliorated iron-triggered follicular deterioration. The 3SH group displayed restored numbers of primordial, primary, and secondary follicles alongside a notable reduction in atretic follicles (*p* < 0.05). Similarly, the 6SH group presented an increased abundance of primordial follicles and corpora lutea, while the atretic follicle count was distinctly decreased (*p* < 0.05; Figure 3A,B). Transmission electron microscopy (TEM) revealed typical ferroptosis-associated mitochondrial alterations in granulosa cells of the HFe group, including mitochondrial shrinkage and outer membrane disruption (Figure 3C). Conversely, ameliorated mitochondrial morphology and enhanced mitochondrial autophagy were observed in granulosa cells of the 6SH and 3SH groups, respectively (Figure 3C).

**Figure 4:**
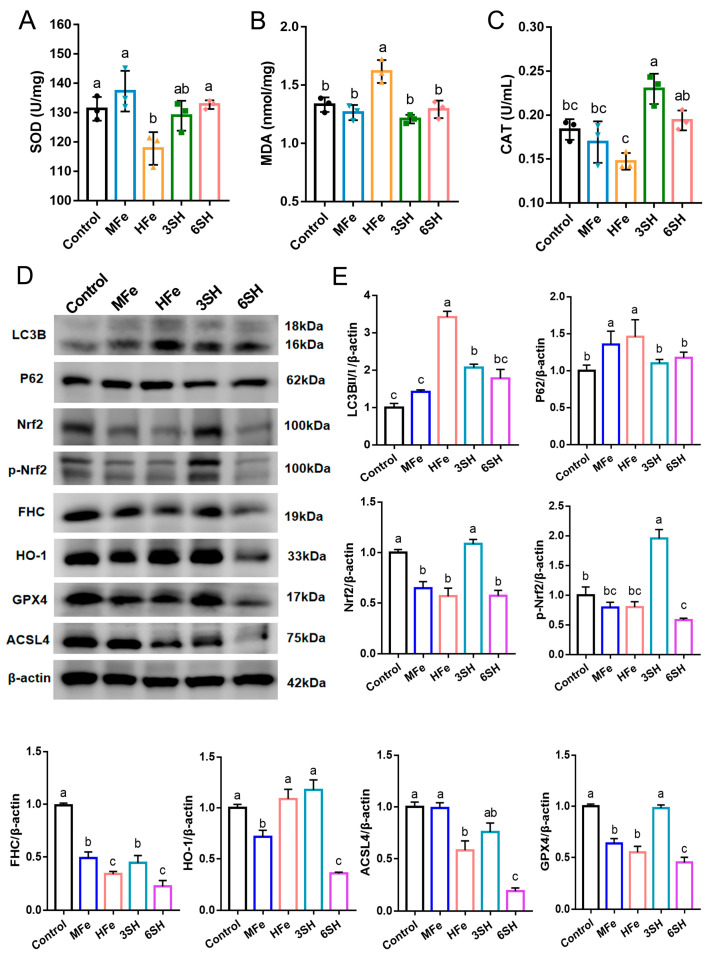
Effects of spermidine on oxidative stress indexes and ferroptosis-related protein expression in iron overloaded mouse ovaries. (**A**) SOD activity, (**B**) MDA content, (**C**) CAT activity in ovarian tissues. (**D**) Western blotting analysis of ferroptosis and autophagy-related protein expression in ovarian tissues. (**E**) Data are presented as mean ± SD, (*n* = 3), same letters indicate no significant difference (*p* > 0.05), different letters indicate significant difference (*p* < 0.05).

Oxidative stress biomarker analysis revealed alterations in ovarian antioxidant status across groups. Compared with the HFe group, both the 3SH and 6SH groups exhibited markedly enhanced ovarian CAT activity (*p* < 0.05). The 6SH group also showed a significant elevation in ovarian SOD activity (*p* < 0.05), while MDA content was distinctly reduced in both spermidine-treated groups (*p* < 0.05; Figure 4A–C).

Western blotting further indicated that relative to the HFe group, the 3SH group presented significant upregulation of Nrf2, p-Nrf2, GPX4 and FHC protein levels (*p* < 0.05), accompanied by a remarkable decline in the LC3BII/I ratio (*p* < 0.05). These findings suggested that spermidine supplementation suppressed excessive autophagy in ovarian tissues under iron overload conditions (Figure 4D).

### 3.4. Effects of Ferric Ammonium Citrate (FAC) on Granulosa Cell Viability and Ferroptosis-Related Protein Expression, and the Intervention Effect of Spermidine

**Figure 5:**
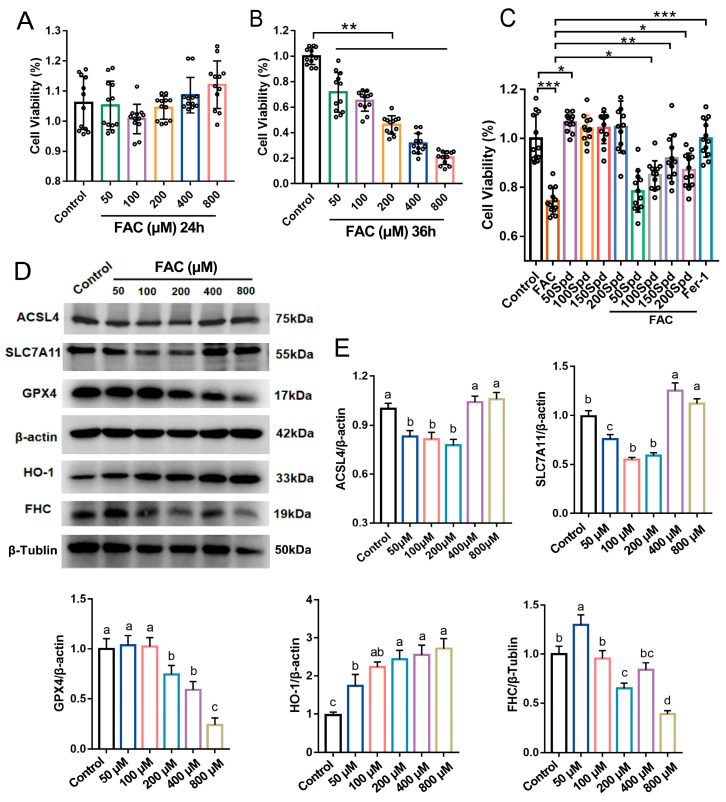
Effects of spermidine (Spd) on FAC-induced granulosa cell viability and ferroptosis-related protein expression. (**A**,**B**) CCK-8 assay to detect the effect of FAC on granulosa cell viability. (**C**) CCK-8 assay to detect the effect of spermidine on FAC-impaired granulosa cell viability. FAC: ferric ammonium citrate, a ferroptosis inducer; Fer-1: ferrostatin-1, a ferroptosis inhibitor. (**D**) Western blotting analysis of ferroptosis-related protein expression in granulosa cells and (**E**) its quantitative analysis. Data are presented as mean ± SD, (*n* = 3). * *p* < 0.05, ** *p* < 0.01, *** *p* < 0.001. Same letters indicate no significant difference (*p* > 0.05), different letters indicate significant difference (*p* < 0.05).

To explore the regulatory role of spermidine in FAC-triggered ferroptosis of granulosa cells, we first screened the optimal concentrations and intervention durations of FAC and spermidine via CCK-8 assay. Cell viability analysis showed that exposure to 200 μM FAC for 24 h exerted no obvious cytotoxicity to porcine granulosa cells (*p* > 0.05; Figure 5A). In contrast, prolonged treatment for 36 h markedly decreased cell viability to approximately 50% relative to the control (*p* < 0.01; Figure 5B). Accordingly, 200 μM FAC treatment for 36 h was chosen as the optimal condition for subsequent in vitro experiments.

Subsequent spermidine intervention assays demonstrated that compared with the FAC-alone group, 100, 150, and 200 μM spermidine markedly restored granulosa cell viability (*p* < 0.05), with 150 μM spermidine exhibiting the most prominent protective effect (*p* < 0.01; Figure 5C). Western blotting further revealed that relative to the control group, 200 μM FAC intervention significantly downregulated the protein levels of FHC, GPX4 and SLC7A11, while distinctly upregulating heme oxygenase-1 (HO-1) protein expression in granulosa cells (*p* < 0.05; Figure 5D,E).

### 3.5. Effects of Spermidine on Lipid Peroxidation and Iron Homeostasis of FAC-Induced Iron Overload Granulosa Cells

**Figure 6:**
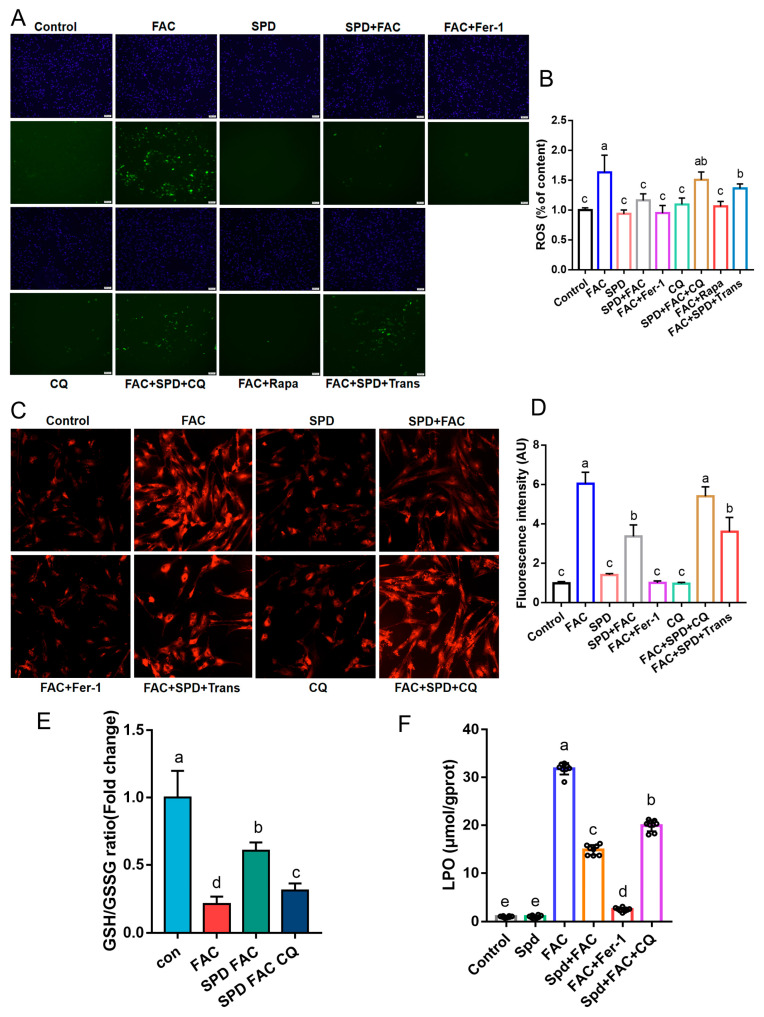
Effects of spermidine on FAC-induced lipid peroxidation and Fe^2+^ levels in granulosa cells. (**A**) Intracellular ROS fluorescence images (green) and (**B**) its quantitative analysis. Blue: DAPI, indicating the nucleus. Scale bar, 200 μm. Fer-1: ferrostatin-1; CQ: chloroquine, an autophagy inhibitor; Rapa: rapamycin, an autophagy inducer; Trans: trans-4-Methylcyclohexyl amine, a spermidine synthase inhibitor. (**C**) Intracellular Fe^2+^ fluorescence images (red) and (**D**) its quantitative analysis. Scale bar, 50 μm. (**E**) Intracellular GSH/GSSG ratio. (**F**) Intracellular lipid peroxidation levels. LPO: lipid peroxidation products. Data are presented as mean ± SD, (*n* = 3), same letters indicate no significant difference (*p* > 0.05), different letters indicate significant difference (*p* < 0.05).

To elucidate the regulatory mechanism of spermidine on lipid peroxidation under iron overload, intracellular ROS accumulation, lipid peroxidation status, and the GSH/GSSG ratio were determined in granulosa cells. Compared with the FAC group, spermidine intervention markedly diminished intracellular ROS abundance and mitigated lipid peroxidation (*p* < 0.05; Figure 6A–C), while effectively elevating the GSH/GSSG ratio (*p* < 0.05; Figure 6E). Rapamycin (Rapa), an autophagy agonist, exerted a comparable protective effect and prominently suppressed FAC-triggered ROS overproduction (*p* < 0.05; Figure 6A,B). FerroOrange staining further demonstrated that 150 μM spermidine efficiently reduced intracellular Fe^2+^ deposition relative to the FAC group (*p* < 0.05; Figure 6C,D). Mechanistically, pharmacological inhibition of spermidine synthase using Trans abolished the ROS-scavenging capacity of spermidine; notably, the FAC + SPD + Trans group exhibited higher ROS levels than the FAC + SPD group (*p* < 0.05). Similarly, chloroquine (CQ)-mediated autophagy inhibition compromised the suppressive effect of spermidine on lipid peroxidation. CQ treatment also increased intracellular ROS and Fe^2+^ accumulation (*p* < 0.05; Figure 6A,B,E,F). Collectively, these data demonstrated that 150 μM spermidine alleviates FAC-induced ROS burst, lipid peroxidation, and intracellular iron overload via autophagy activation, thereby maintaining cellular iron homeostasis and facilitating intracellular GSH synthesis.

### 3.6. Effects of Spermidine on Mitochondrial Morphology and Function of FAC-Induced Iron Overload Granulosa Cells

**Figure 7:**
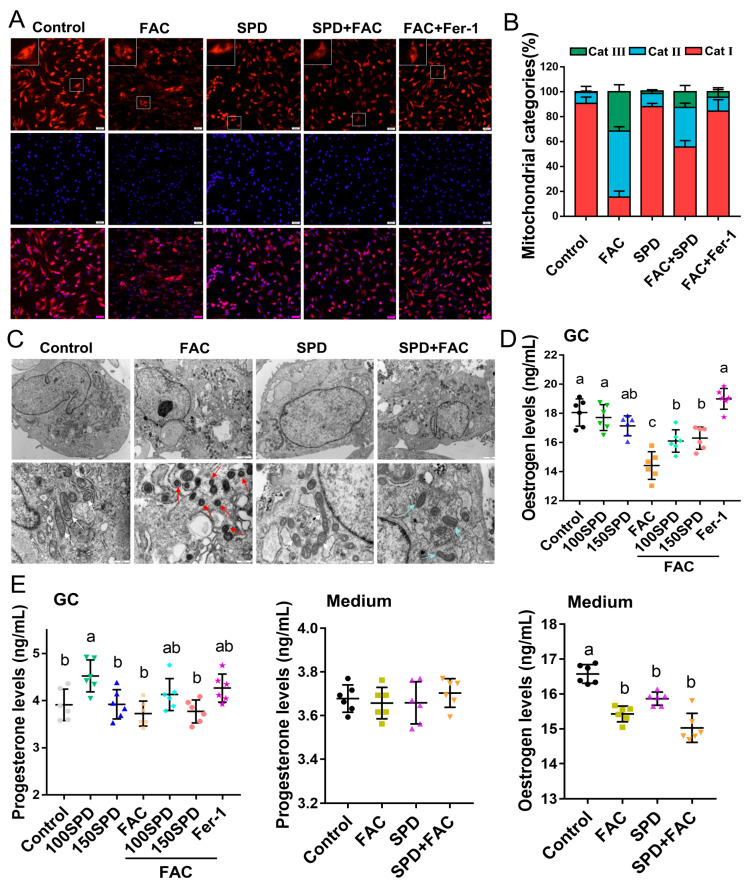
Effects of spermidine on FAC-induced mitochondrial morphological changes in granulosa cells. (**A**) Mitochondrial morphology detected by Mito-Tracker Red staining (Cat I: normal slender reticular mitochondria; Cat III: severe fragmentation with perinuclear mitochondrial debris). Cat: category. In the upper left corner frame is a locally enlarged field of view. Scale bar, 50 μm. (**B**) Quantitative analysis of mitochondrial morphology. (**C**) TEM observation of mitochondrial ultrastructure (white arrow: normal mitochondria; red arrow: ferroptotic mitochondria; blue arrow: spermidine-repaired mitochondria; Scale bar, 2.0 μm [upper] or 1.0 μm [lower]). (**D**) Estradiol levels in granulosa cell lysates and culture supernatants. (**E**) Progesterone levels in granulosa cell lysates and culture supernatants. GC: granulosa cells. Data are presented as mean ± SD, (*n* = 3), same letters indicate no significant difference (*p* > 0.05), different letters indicate significant difference (*p* < 0.05).

Mito-Tracker Red staining was performed to assess mitochondrial morphological characteristics, which were categorized into three grades: Category I (Cat.I, normal slender reticular mitochondria), Category II (Cat.II, mild fragmentation), and Category III (Cat.III, severe fragmentation with perinuclear debris). Mitochondrial morphological analysis revealed that FAC exposure triggered extensive mitochondrial fragmentation in granulosa cells, accompanied by a prominently elevated proportion of Cat.III mitochondria (*p* < 0.01). Conversely, spermidine intervention effectively reversed such ferroptosis-associated mitochondrial damage; the SPD-treated group exhibited an increased percentage of Cat.I mitochondria and a decreased proportion of Cat.III mitochondria relative to the FAC group (*p* < 0.05; Figure 7A,B). Consistent with these findings, TEM observation identified typical ferroptotic mitochondrial alterations in FAC-exposed granulosa cells, including mitochondrial shrinkage, enhanced membrane density, cristae reduction or loss, and outer membrane rupture (red arrows; Figure 7C). Notably, these detrimental morphological abnormalities were markedly ameliorated in the FAC + SPD group, where mitochondrial structure was largely recovered (blue arrows; Figure 7C). Hormonal measurement further demonstrated that 100 and 150 μM spermidine significantly elevated estradiol concentrations in granulosa cell lysates compared with the FAC group (*p* < 0.05; Figure 7D), whereas no obvious variations in estradiol and progesterone levels were detected in the culture supernatant (*p* > 0.05; Figure 7E). Collectively, these observations indicated that spermidine facilitates intracellular estrogen synthesis in iron-overloaded granulosa cells without affecting extracellular hormonal secretion.

### 3.7. Effects of Spermidine on Mitochondrial Membrane Potential, Ferroptosis-Related Proteins and DNA Damage in FAC-Induced Iron Overload Granulosa Cells

**Figure 8:**
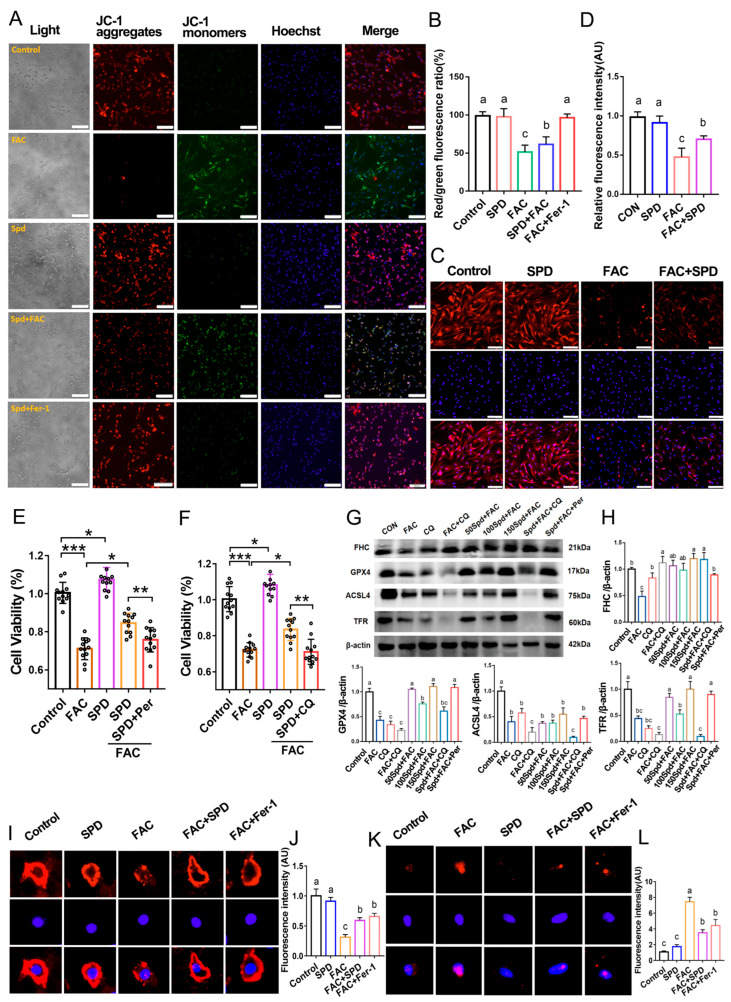
Effects of spermidine on mitochondrial membrane potential, ferroptosis-related proteins and DNA damage in FAC-induced iron overload granulosa cells. (**A**) Mitochondrial membrane potential detected by JC-1 staining and (**B**) its quantitative analysis (red/green fluorescence ratio, *n* = 3). Scale bar, 100 μm. (**C**) GPX4 immunofluorescence staining (red) and (**D**) its quantitative analysis. Scale bar, 50 μm. (**E**,**F**) CCK-8 assay to detect the effect of Per (Perifosine, an AKT inhibitor) and CQ (chloroquine, an autophagy inhibitor) on spermidine-protected granulosa cell viability. (**G**) Western blotting analysis of ferroptosis-related protein expression and (**H**) its quantitative analysis. (**I**) AMH immunofluorescence staining (red) and (**J**) its quantitative analysis. Scale bar, 2 μm. (**K**) γH2AX immunofluorescence staining (red) and (**L**) its quantitative analysis. Scale bar, 2 μm. Data are presented as mean ± SD, (*n* = 3). * *p* < 0.05, ** *p* < 0.01, *** *p* < 0.001, same letters indicate no significant difference (*p* > 0.05), different letters indicate significant difference (*p* < 0.05).

MMP measurement revealed that spermidine intervention effectively increased the JC-1 red/green fluorescence ratio relative to the FAC group (*p* < 0.05), suggesting the restorative effect of spermidine on mitochondrial membrane potential in iron-overloaded granulosa cells (Figure 8A,B). MitoSOX Red staining further confirmed that spermidine remarkably suppressed FAC-triggered mitochondrial superoxide accumulation (Supplementary Figure S1). Western blotting and immunofluorescence analyses demonstrated that the FAC + SPD group exhibited pronounced upregulation of the ferroptosis-related protein GPX4 and the ovarian reserve marker anti-müllerian hormone (AMH) (*p* < 0.05; Figure 8C,D,I,J), while the expression of the DNA damage marker gamma histone family 2A variant X (γH2AX) was distinctly downregulated (*p* < 0.05; Figure 8K,L). Additionally, spermidine supplementation markedly elevated the protein levels of FHC, GPX4, and TfR1 in FAC-exposed granulosa cells (*p* < 0.05; Figure 8G,H).

Further functional inhibition experiments were conducted to clarify the underlying regulatory mechanism. Pharmacological blockade of autophagy using chloroquine (CQ) reduced cell viability in the FAC + SPD + CQ group (*p* < 0.05; Figure 8F) and concurrently downregulated GPX4 and TfR1 expression compared with the FAC + SPD group (*p* < 0.05; Figure 8G,H). In addition, perifosine-mediated inhibition of the PI3K-AKT pathway also decreased cell viability in the FAC + SPD + Per group (*p* < 0.05; Figure 8E). Notably, PI3K-AKT inhibition exerted no significant influence on the protein abundances of FHC, GPX4, and TfR1 (*p* > 0.05; Figure 8G,H). Collectively, these findings illustrated that spermidine ameliorates FAC-induced granulosa cell ferroptosis through multiple regulatory pathways. Mechanistically, spermidine improves mitochondrial morphology and MMP, inhibits mitochondrial ROS burst and DNA damage, and elevates the expression levels of FHC, GPX4, TfR1, and AMH. Furthermore, the cytoprotective effects of spermidine are dependent on both autophagy activation and PI3K-AKT signaling initiation.

### 3.8. Spermidine Up-Regulates FHC to Regulate Iron Homeostasis and GSH Levels in Granulosa Cells

**Figure 9:**
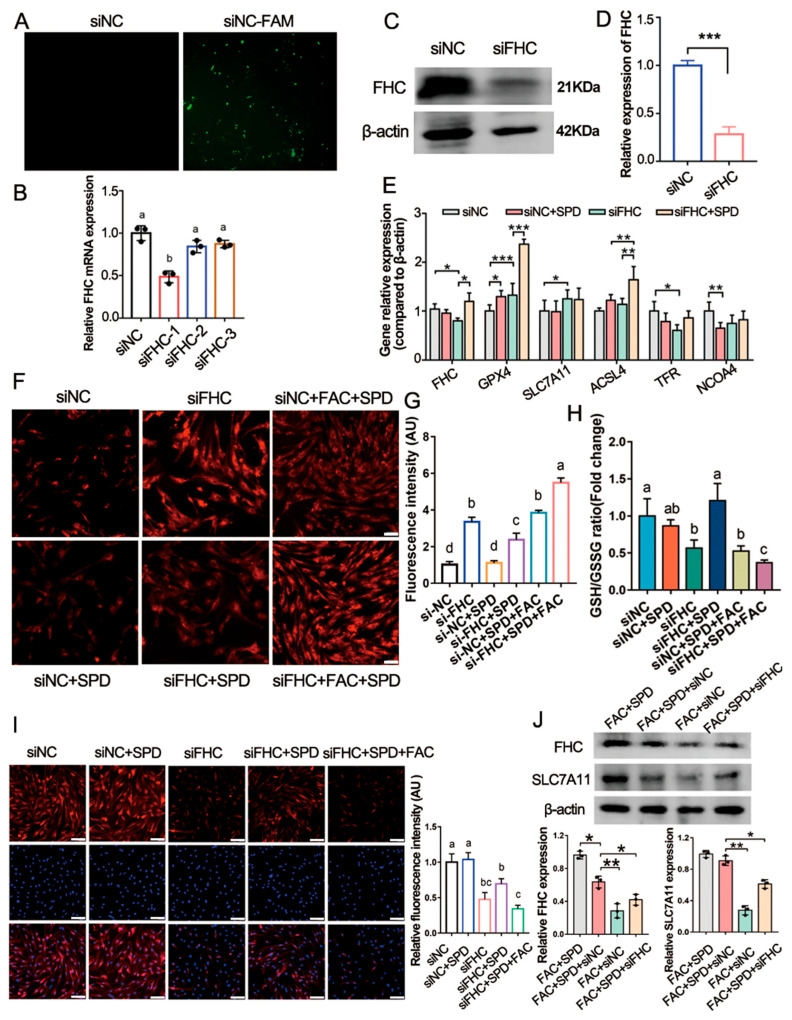
Spermidine inhibits ferroptosis by upregulating FHC to regulate iron and GSH levels in granulosa cells. (**A**) Fluorescence images of siRNA-FHC-transfected granulosa cells (green, 48 h). Scale bar, 200 μm. (**B**) qPCR analysis of the interference efficiency of three FHC siRNAs. (**C**) FHC protein expression detected by Western blotting and (**D**) its quantitative analysis (72 h post-transfection, *n* = 3). (**E**) qPCR detection of ferroptosis-related gene expression. (**F**) Fe^2+^ fluorescence images detected by FerroOrange staining (red) and (**G**) its quantitative analysis. Scale bar, 50 μm. (**H**) Intracellular GSH levels. (**I**) GPX4 immunofluorescence staining (red) and its quantitative analysis. Scale bar, 50 μm. (**J**) Western blotting analysis of ferroptosis-related protein expression (*n* = 3). * *p* < 0.05, ** *p* < 0.01, *** *p* < 0.001, same letters indicate no significant difference (*p* > 0.05), different letters indicate significant difference (*p* < 0.05).

**Figure 10:**
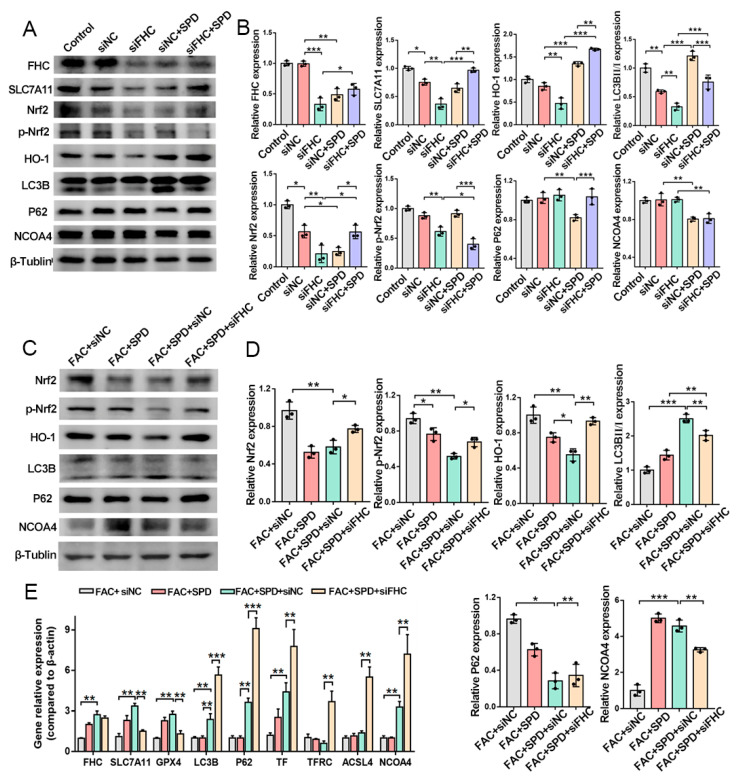
Effects of spermidine on FAC-induced ferroptosis and autophagy in FHC-silenced granulosa cells. (**A**) Western blotting analysis of ferroptosis and autophagy-related protein expression and (**B**) its quantitative analysis. (**C**) Western blotting analysis of ferroptosis and autophagy-related protein expression under FAC-induced iron overload and (**D**) its quantitative analysis. (**E**) qPCR detection of ferroptosis and autophagy-related gene expression under FAC-induced iron overload. Data are presented as mean ± SD, (*n* = 3). * *p* < 0.05, ** *p* < 0.01, *** p < 0.001.

To elucidate the regulatory role of FHC in the anti-ferroptotic capacity of spermidine, FHC expression was genetically silenced in granulosa cells via siRNA transfection. Three distinct FHC siRNA sequences were designed, and transfection efficiency was validated using qPCR and Western blotting. The results indicated that siRNA-FHC1 exhibited the highest knockdown efficiency. Specifically, siRNA-FHC1 markedly reduced *FHC* mRNA abundance at 48 h (*p* < 0.01; Figure 9A,B) and decreased FHC protein accumulation at 72 h (*p* < 0.01; Figure 9C,D) relative to the negative control (siNC), thereby confirming successful FHC suppression. FHC knockdown significantly elevated intracellular Fe^2+^ levels (*p* < 0.05; Figure 9F,G), diminished intracellular GSH content (*p* < 0.05; Figure 9H), and downregulated *TfR1* mRNA expression (*p* < 0.05; Figure 9E). Of note, spermidine intervention effectively reversed these FHC-deficiency-induced abnormalities in siFHC-transfected granulosa cells. Spermidine treatment reduced excessive intracellular Fe^2+^ deposition (*p* < 0.05; Figure 9F,G) and increased the mRNA and protein levels of FHC, GSH, and GPX4 (*p* < 0.05; Figure 9E,I and Figure 10A). These data demonstrated that spermidine alleviates intracellular iron overload and enhances antioxidant capacity by upregulating FHC expression.

Western blotting further revealed that FHC silencing suppressed the protein abundances of SLC7A11, Nrf2/p-Nrf2/HO-1, and microtubule-associated protein 1 light chain 3 beta (LC3B), while increasing sequestosome 1 (P62) protein accumulation (*p* < 0.05; Figure 10A,B). Conversely, spermidine supplementation counteracted these molecular alterations in siFHC cells; spermidine restored the protein expression of Nrf2/HO-1 and LC3B and decreased nuclear receptor coactivator 4 (NCOA4) protein levels (*p* < 0.05; Figure 10A,B). Under FAC-mediated iron overload conditions, the FAC + SPD + siNC group exhibited downregulated protein levels of Nrf2/p-Nrf2/HO-1 and P62, alongside elevated FHC, SLC7A11, and LC3B expression compared with the FAC + siNC group (*p* < 0.05; Figure 10C,D). However, FHC depletion abolished the protective effects of spermidine. The FAC + SPD + siFHC group displayed enhanced protein expression of Nrf2/p-Nrf2/HO-1 and P62, accompanied by reduced abundances of SLC7A11, LC3B, and NCOA4 (*p* < 0.05; Figure 10C,D).

Consistent with the protein results, qPCR analysis showed that spermidine remarkably upregulated the mRNA levels of *FHC*, *SLC7A11*, *GPX4*, *LC3B*, *P62*, and *TfR1* in iron-overloaded granulosa cells (FAC + SPD + siNC vs. FAC + siNC; *p* < 0.05; Figure 10E). In contrast, FHC knockdown inhibited the transcriptional expression of *SLC7A11* and *GPX4*, whereas it further increased the mRNA abundances of *LC3B*, *P62*, *ACSL4*, and *NCOA4* (FAC + SPD + siFHC vs. FAC + SPD + siNC; *p* < 0.05; Figure 10E). Collectively, these findings indicated that spermidine facilitates autophagy activation and upregulates FHC and LC3B expression at both transcriptional and translational levels. This modulation maintains cellular iron homeostasis and GSH synthesis and restrains excessive activation of the Nrf2/p-Nrf2/HO-1 pathway during ferroptosis. Notably, FHC deficiency triggered compensatory reactivation of the antioxidant Nrf2/p-Nrf2/HO-1 cascade and suppressed autophagic progression in granulosa cells.

### 3.9. Spermidine Activates Autophagy via the AKT Pathway and Upregulates SLC7A11 to Improve Mitochondrial Iron Homeostasis in Granulosa Cells

**Figure 11:**
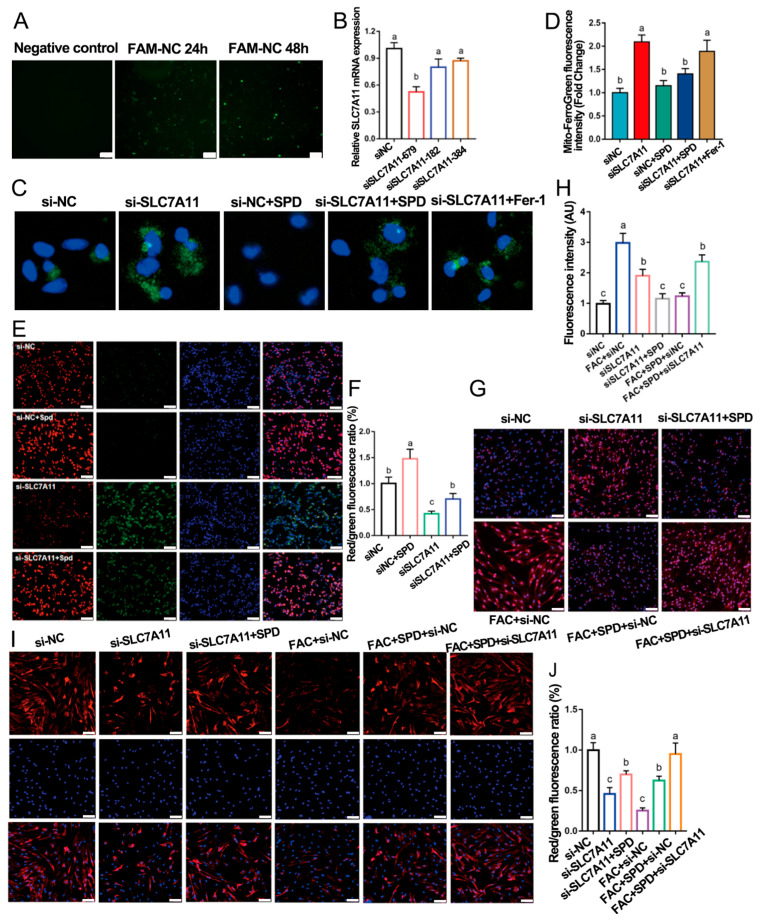
Spermidine upregulates SLC7A11 to improve mitochondrial function in granulosa cells. (**A**) Green fluorescence of si-SLC7A11-transfected cells at 24 h and 48 h. Scale bar, 200 μm. (**B**) qPCR analysis of interference efficiency for three SLC7A11 siRNAs. (**C**) Mitochondrial Fe^2+^ staining (green fluorescence) in SLC7A11-silenced cells and (**D**) quantitative analysis. Scale bar, 2 μm. (**E**) Effects of spermidine on mitochondrial membrane potential in SLC7A11-knockdown cells and (**F**) quantitative analysis (red/green fluorescence ratio). Scale bar, 100 μm. (**G**) Effects of spermidine on mitochondrial superoxide levels (red fluorescence) in SLC7A11-silenced cells and (**H**) quantitative analysis. Scale bar, 50 μm. (**I**) SLC7A11 immunofluorescence staining (red) and (**J**) quantitative analysis (*n* = 3). Scale bar, 50 μm. Same letters indicate no significant difference (*p* > 0.05), different letters indicate significant difference (*p* < 0.05).

Previous evidence has demonstrated that FHC modulates SLC7A11 activity by regulating cystine/glutamate exchange, thereby protecting cells against lipid peroxidation-mediated ferroptotic death [26]. To further clarify whether spermidine ameliorates ferroptosis in an SLC7A11-dependent manner, SLC7A11 expression was genetically suppressed in granulosa cells via siRNA transfection. Three distinct SLC7A11 siRNA sequences were constructed, and qPCR validation confirmed that siSLC7A11-679 exerted the highest knockdown efficiency. This fragment markedly decreased *SLC7A11* mRNA abundance at 48 h post-transfection (*p* < 0.01; Figure 11A,B), indicating successful SLC7A11 silencing.

Mito-FerroGreen staining showed that SLC7A11 depletion triggered mitochondrial iron accumulation and mitochondrial oxidative stress. Relative to the siNC group, the siSLC7A11 group exhibited elevated mitochondrial Fe^2+^ deposition and increased mitochondrial superoxide generation, accompanied by impaired mitochondrial membrane potential (MMP) (Figure 11C–H). Notably, spermidine intervention effectively reversed these mitochondrial abnormalities in SLC7A11-deficient cells. The siSLC7A11 + SPD group displayed diminished mitochondrial Fe^2+^ and ROS levels alongside restored MMP. Collectively, these phenotypic observations indicated that spermidine improves mitochondrial iron homeostasis by upregulating SLC7A11, thereby mitigating mitochondrial iron overload, reducing oxidative burst, and preserving mitochondrial function.

**Figure 12:**
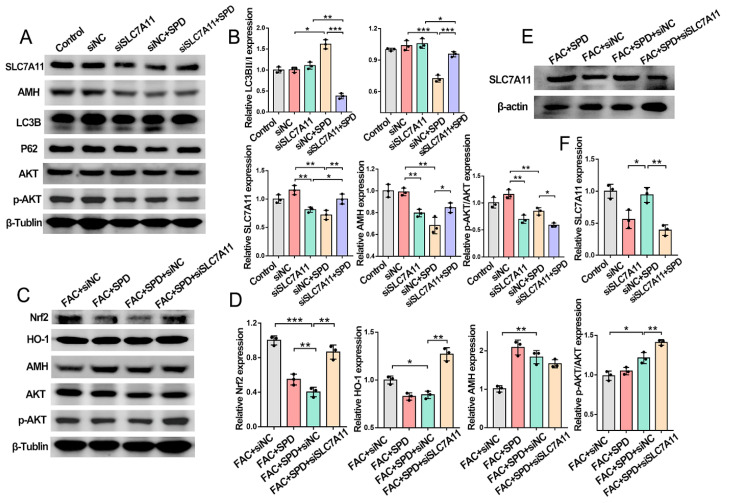
Effect of spermidine on autophagy, antioxidant capacity and ovarian reserve in SLC7A11-silenced granulosa cells. (**A**,**B**) Effects of spermidine on autophagy, AMH levels and the AKT pathway. (**C**–**F**) Regulatory effects of spermidine on antioxidant capacity, ovarian reserve and the AKT pathway under ferroptotic conditions (*n* = 3). * *p* < 0.05, ** *p* < 0.01, *** *p* < 0.001.

Immunofluorescence and Western blotting analyses further validated the molecular regulatory relationship between spermidine and SLC7A11. SLC7A11 knockdown reduced the protein abundances of SLC7A11, AMH, and p-AKT/AKT, whereas spermidine supplementation partially restored SLC7A11 expression (Figure 11I,J and Figure 12A,B). Moreover, compared with the siNC + SPD group, the siSLC7A11 + SPD group presented decreased LC3B and p-AKT/AKT levels while showing increased P62 accumulation. These results suggested that SLC7A11 deficiency impedes spermidine-mediated autophagy activation and AKT signaling phosphorylation.

Under FAC-induced iron-overload conditions, spermidine treatment distinctly upregulated the protein expression of SLC7A11, AMH, and p-AKT/AKT and suppressed Nrf2 and HO-1 abundances (FAC + SPD + siNC vs. FAC + siNC; Figure 12C–F). Conversely, SLC7A11 silencing abolished the inhibitory effects of spermidine on the Nrf2/HO-1 cascade; the FAC + SPD + siSLC7A11 group exhibited further elevated Nrf2/HO-1 and p-AKT/AKT protein levels relative to the FAC + SPD + siNC group.

Collectively, these findings demonstrated that spermidine triggers autophagic activation and facilitates SLC7A11 upregulation via AKT signaling modulation, thereby maintaining mitochondrial iron homeostasis in granulosa cells. SLC7A11 depletion exacerbates mitochondrial ROS deposition and blocks autophagic progression, ultimately disrupting granulosa cell iron metabolism.

### 3.10. Proteomic Sequencing Identifies Key Proteins and Enrichment Pathways After SLC7A11 Silencing in Granulosa Cells

**Figure 13:**
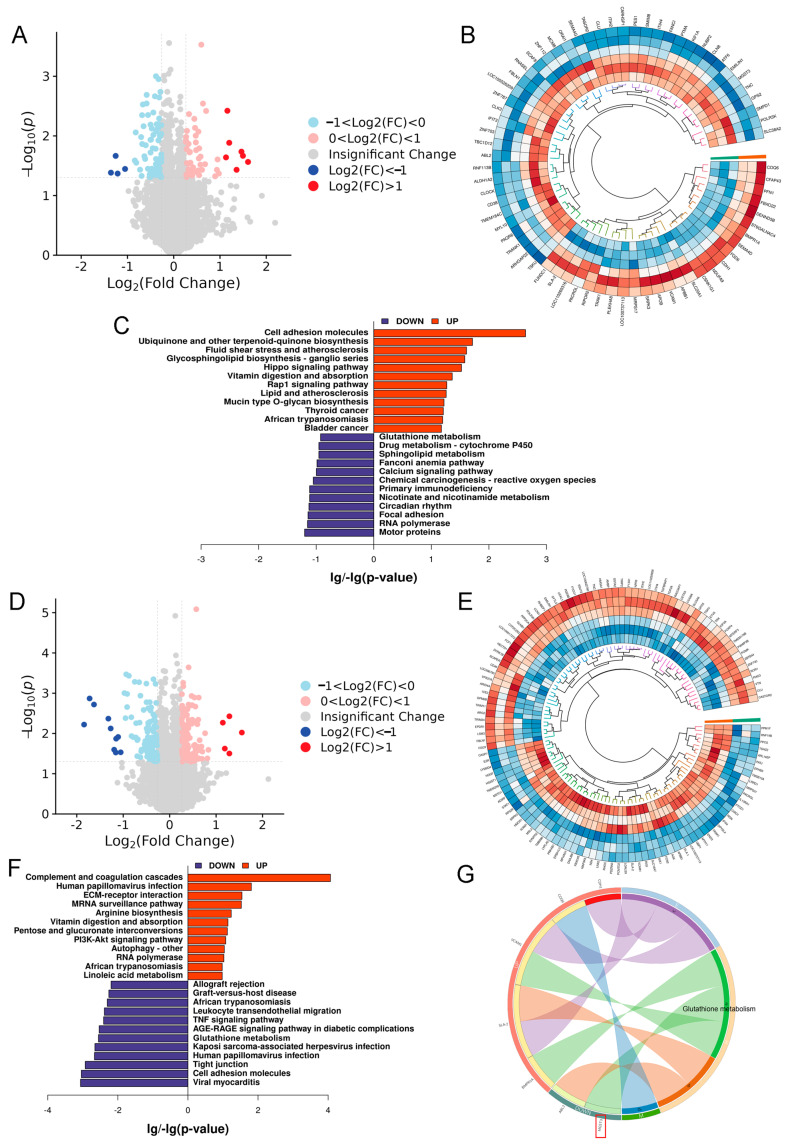
Screening of differentially expressed proteins and their enriched signaling pathways. (**A**–**C**) Comparison between the FAC and FAC + SPD groups. (**D**–**G**) Comparison between the FAC + SPD and FAC + SPD + siSLC7A11 groups. (**A**,**D**) Volcano plots of differentially expressed proteins. (**B**,**E**) Cluster heatmaps of differentially expressed proteins. Red indicates up-regulation, while blue indicates down-regulation. (**C**,**F**) KEGG pathway enrichment analysis of differentially expressed proteins. (**G**) String interaction network between differentially expressed proteins and related signaling pathways. Left half of the string graph: proteins (from outer to inner: gene symbol, expression trend, log2(fold change)). Right half of the string graph: a–d represents different pathways (from outer to inner: pathway category, pathway name).

To further explore the downstream molecular mechanism by which spermidine alleviates ferroptosis via SLC7A11, TMT-based quantitative proteomic sequencing was performed. Volcano plot analysis identified 133 differentially expressed proteins (DEPs) between the FAC and FAC + SPD groups (Figure 13A), while 236 DEPs were screened between the FAC + SPD and FAC + SPD + siSLC7A11 groups (Figure 13D). Cluster heatmap analysis revealed evident intergroup separation and high intragroup consistency, confirming reliable sample grouping and satisfactory biological reproducibility (Figure 13B,E). KEGG enrichment analysis indicated that the DEPs derived from the FAC versus FAC + SPD comparison were primarily enriched in the Hippo, Rap1, calcium signaling, and glutathione metabolism pathways (Figure 13C). In contrast, DEPs identified in the FAC + SPD versus FAC + SPD + siSLC7A11 comparison were mainly associated with the PI3K-AKT signaling pathway, autophagy, glutathione metabolism, and the TNF signaling cascade (Figure 13F).

As a critical cystine/glutamate antiporter, SLC7A11 fundamentally modulates glutathione synthesis and intracellular oxidative status. SLC7A11 silencing impeded cystine uptake, suppressed GSH biosynthesis, disrupted glutathione metabolic homeostasis, triggered excessive ROS accumulation, and impaired autophagic progression, which was consistent with the molecular phenotypic changes presented in Figure 12. Further bioinformatic screening identified microsomal glutathione S-transferase 3 (MGST3) as a core differentially expressed protein involved in glutathione metabolism (Figure 13G). As a nuclear-encoded mitochondrial enzyme, MGST3 may act as a vital downstream effector of the spermidine–SLC7A11 axis to antagonize granulosa cell ferroptosis.

## 4. Discussion

In this study, we demonstrate for the first time that spermidine alleviates iron overload-induced ovarian dysfunction by activating the FHC/SLC7A11 axis to regulate iron homeostasis and inhibit granulosa cell ferroptosis, and we further identify MGST3 as a potential downstream target of this signaling axis. In women, serum iron levels increase with age, accompanied by ovarian dysfunction and menstrual irregularities, due to impaired excess iron excretion [27,28]. Ferroptosis has been recognized as a key driver of ovarian dysfunction. The activity of key enzymes involved in ovarian steroidogenesis is dependent on electron transfer, and ferritin participates in the regulation of this process. Therefore, iron is indispensable for ovarian hormone secretion [29].

In our previous study, drinking water supplementation with 3 mM spermidine reduced the number of atretic follicles in female ICR mice under normal physiological conditions [30]. It protected ovarian function by activating ovarian autophagy, enhancing antioxidant enzyme activity and upregulating polyamine metabolism levels. In addition, 24 h treatment of goose follicular granulosa cells with 160 μM spermidine could induce autophagy, thereby alleviating oxidative stress and cell apoptosis [31]. Therefore, in the present study, we adopted 3 mM spermidine via drinking water to treat ICR mice, and applied spermidine at concentrations ranging from 50 to 200 μM to culture porcine follicular granulosa cells in vitro.

Our results showed that the estrous cycle was severely disrupted in HFe mice (Figure 2A,B), which may be attributed to decreased estrogen levels (Figure 2D). Treatment with 3 mM spermidine increased the proportion of regular estrous cycles and ovarian index (Figure 1D), elevated serum estradiol and progesterone levels (Figure 2D,E), reduced iron ion levels, and restored iron homeostasis in serum and ovarian tissues (Figure 1F,G). In the present study, cytoplasmic FHC expression gradually decreased with increasing dietary iron concentration (Figure 4D, *p* < 0.05). These iron overload-induced ovarian impairments were significantly ameliorated by spermidine treatment, suggesting that the protective effect of spermidine relies on its ability to regulate iron homeostasis and suppress ferroptosis.

Consistent with previous studies reporting that excessive iron intake causes reproductive toxicity [32], our data confirmed that high iron intake disrupted estrous cycles and reduced ovarian hormone levels in mice (Figure 2). Gokce et al. [33] measured spermidine levels in follicular fluid from patients with diminished ovarian reserve (DOR) and found that follicular fluid spermidine levels were significantly higher in the DOR group than in the control group, implying that follicular spermidine may be involved in ovarian aging. Spermidine improves the quality of post-ovulatory aged porcine oocytes by enhancing mitochondrial function, scavenging excessive ROS, inhibiting apoptosis, and promoting autophagy [34].

In accordance with these findings [30], our study showed that spermidine increased the number of healthy follicles, reduced follicular atresia (Figure 3A,B), improved granulosa cell mitochondrial morphology (Figure 3C), inhibited excessive autophagy, activated the Nrf2/p-Nrf2/GPX4 antioxidant pathway, and upregulated the ferroptosis-inhibitory proteins GPX4 and FHC to protect against iron overload-induced ovarian damage (Figure 4).

Within the antioxidant system, FHC maintains iron bioavailability and catalytic inactivation, prevents free iron accumulation in the labile iron pool (LIP), and blocks ROS production via the Fenton reaction. In the present study, TfR1 and FHC levels were decreased in iron-overloaded granulosa cells (Figure 8G,H), indicating that iron overload disrupts iron homeostasis by increasing iron influx and decreasing iron sequestration. Salatino et al. [35] reported that FHC is essential for the normal function of the antioxidant system, and FHC inhibition enhances cisplatin-induced cytotoxicity. Liu et al. [36] demonstrated that high cytoplasmic FHC expression predicts a favorable disease prognosis. When TfR1 expression is significantly upregulated, cells maintain iron homeostasis and reduce free iron accumulation by increasing FHC expression [37,38].

Our results confirmed that spermidine alleviates FAC-induced ferroptosis in granulosa cells, upregulates the ferroptosis-inhibitory proteins FHC, GPX4, and TfR1 as well as the ovarian reserve marker AMH (Figure 8A–J), and suppresses mitochondrial ROS production (Figure S1) and DNA damage (Figure 8K,L). Additionally, spermidine significantly increased estradiol synthesis in granulosa cells but had no significant effect on progesterone levels (Figure 7D,E).

Ferritinophagy is a key mechanism underlying reduced intracellular FHC levels. Inhibition of lysosomal function blocks FHC degradation, reduces iron accumulation and lipid peroxidation, and attenuates ferroptosis [39]. Scaramuzzino et al. [40] found that FHC knockout increases apoptosis and DNA damage in embryonic stem cells, decreases total ROS levels, and triggers excessive activation of the p62–Keap1–Nrf2 pathway. In this study, FHC silencing significantly increased intracellular free Fe^2+^ levels, and spermidine treatment reversed this effect (Figure 9F,G).

Spermidine activated autophagy, promoted FHC expression to regulate iron homeostasis and GSH levels, and attenuated excessive activation of the Nrf2/p-Nrf2/HO-1 antioxidant pathway under ferroptotic conditions. Our in vitro data confirmed that spermidine upregulated FHC expression, which chelated intracellular free Fe^2+^, reduced the labile iron pool, inhibited the Fenton reaction, and decreased ROS production (Figure 6). When FHC was knocked down, the Nrf2/p-Nrf2/HO-1 pathway was reactivated as a compensatory mechanism to counteract FHC deficiency-induced dysfunction, and cellular autophagy was suppressed (Figure 10C,D), which is consistent with previous reports [39].

Cheng et al. [41] reported that spermidine inhibits NCOA4-mediated ferritinophagy, restores the expression of the key antioxidant proteins SLC7A11 and GPX4, suppresses lipid peroxidation and iron metabolism disorders, and protects chondrocytes against IL-1β-induced ferroptosis. Studies have also shown that spermidine promotes placental angiogenesis and tissue growth in sows, increases the number of healthy piglets and placental efficiency, upregulates amino acid and glucose transporters (SLC7A7, SLC2A2, SLC5A4) in placental tissue, and improves reproductive performance [42]. Spermidine upregulates SLC7A11, glutamate cysteine ligase modifier subunit (GCLM), and GPX4 expression through ATF4 activation, enhances cell viability, and alleviates mitochondrial dysfunction and oxidative stress induced by free fatty acids [43].

In line with these findings, our study demonstrated that spermidine upregulates SLC7A11 expression, reduces mitochondrial Fe^2+^ and superoxide levels, increases mitochondrial membrane potential, and improves mitochondrial function in granulosa cells (Figure 11). The core function of SLC7A11 is to mediate cystine/glutamate reverse transport, and its downstream signaling mainly regulates GSH synthesis and oxidative stress [44]. Inhibition of SLC7A11 caused insufficient cystine uptake, blocked GSH synthesis, induced glutathione metabolic imbalance, and triggered ROS accumulation, which was consistent with our results.

These results indicated that spermidine activates autophagy through the AKT signaling pathway and upregulates SLC7A11 to improve mitochondrial iron homeostasis in granulosa cells (Figure 11C,D and Figure 12A,E). SLC7A11 silencing leads to mitochondrial ROS accumulation (Figure 11G,H) and autophagy inhibition (Figure 12A,B). Our results clearly demonstrated that AKT activation negatively regulates autophagy in granulosa cells, and spermidine regulates autophagy by inhibiting AKT signaling. Compared with the siNC group, the siNC + SPD group shows upregulated LC3B, downregulated P62 and inhibited p-AKT/AKT pathway, indicating spermidine-induced AKT inhibition promotes autophagy (Figure 12A,B). Further SLC7A11 knockdown show that compared with the siNC + SPD group, the siNC + SPD + siSLC7A11 group has downregulated LC3B, upregulated P62 and further inhibited p-AKT/AKT pathway, confirming AKT inhibition is necessary for spermidine to promote autophagy, while autophagy maintenance relies on normal SLC7A11 function.

We found that spermidine mediates protective autophagy rather than pro-ferroptotic autophagy (such as ferritinophagy) in granulosa cells. Spermidine-induced autophagy is accompanied by increased granulosa cell antioxidant capacity, decreased ROS and lipid peroxidation, and inhibited ferroptosis (Figure 6), indicating it exerts a protective effect consistent with protective autophagy. Autophagy activation is positively correlated with ferroptosis inhibition: spermidine-induced autophagy inhibits ferroptosis, while FHC or SLC7A11 knockdown-induced autophagy weakening attenuates this inhibition (Figure 9, Figure 10 and Figure 11 and Figure 12A,B); this differs from pro-ferroptotic effect of ferritinophagy. Regarding the AKT–autophagy–ferroptosis axis, spermidine inhibits AKT to promote protective autophagy, which further enhances antioxidant capacity, reduces ROS and lipid peroxidation, and inhibits ferroptosis, confirming that this autophagy is protective and key to spermidine’s anti-ferroptotic effect.

The porcine granulosa cell model is highly relevant to human reproductive health. Pigs share substantial similarities with humans in ovarian physiology, follicular development, lipid metabolism, and the biological characteristics of granulosa cells. As an established large-animal model, it exhibits greater translational relevance to human reproductive physiology than rodent models. The in vitro findings from porcine ovarian granulosa cells were consistent with the in vivo observations in iron-overloaded female mice. In both experimental models, spermidine markedly reduced iron accumulation, increased estradiol levels, and ameliorated the aberrant mitochondrial morphology induced by iron overload. Moreover, spermidine promoted follicular development in mouse ovarian tissue and restored the viability of iron-injured porcine granulosa cells. Mechanistically, spermidine strengthened antioxidant defenses by elevating SOD and CAT activities, reduced ROS accumulation and lipid peroxidation, and upregulated the expression of FHC and GPX4 to preserve cellular iron homeostasis and inhibit ferroptosis. Furthermore, spermidine regulated AKT-mediated autophagy and normalized the overactivated Nrf2/p-Nrf2/HO-1 signaling cascade. Collectively, these consistent phenotypic and molecular data demonstrate that the protective mechanism of spermidine against iron overload-induced ovarian damage and granulosa cell ferroptosis was conserved between murine and porcine models.

Proteomic analysis revealed that MGST3, a nuclear-encoded mitochondrial protein involved in glutathione metabolism, is a key downstream effector of spermidine-mediated SLC7A11 upregulation and ferroptosis inhibition (Figure 13G). However, the precise regulatory mechanism of MGST3 in the spermidine/SLC7A11-mediated glutathione metabolism and ferroptosis inhibition pathway requires further investigation, as does its potential as a therapeutic target for iron overload-induced infertility. As a nuclear-encoded mitochondrial protein primarily localized in mitochondrial and endoplasmic reticulum membranes, MGST3 may participate in intracellular GSH metabolism by catalyzing GSH conjugation and reducing lipid hydroperoxides, which might contribute to maintaining cellular redox homeostasis [45]. The cystine/glutamate antiporter (Xc^−^ system), with SLC7A11 as its core subunit, is crucial for ferroptosis defense by mediating cystine uptake and supporting GPX4 activity [44]. The specific interaction between MGST3 and SLC7A11 may be the key link through which spermidine inhibits iron overload-induced ferroptosis, with relevant research suggesting that this process may involve three potential mechanisms: Firstly, MGST3 may specifically bind to SLC7A11 via its conserved G-site and hydrophobic motifs, forming a stable complex that could prevent SLC7A11 ubiquitination and degradation, thereby potentially enhancing its membrane localization and cystine transport capacity [46]. Secondly, MGST3 might regulate SLC7A11 function through GSH metabolism, where its catalyzed GSH conjugates may act as specific allosteric activators of SLC7A11, while its GSH-consuming process could activate the Nrf2 pathway to upregulate SLC7A11 transcription [47]. Thirdly, MGST3 may serve as a downstream mediator of spermidine, which might promote MGST3 translocation to the cell membrane, strengthen its binding with SLC7A11, and further enhance SLC7A11 activity to inhibit ferroptosis [48]. All in all, MGST3 may specifically interact with SLC7A11 relying on its structural characteristics and potential GSH metabolic function, which could mediate spermidine’s anti-ferroptotic effect. This hypothesis may help to clarify the regulatory mechanism of spermidine in ovarian iron overload injury and provide a potential therapeutic target for related ovarian disorders.

We established iron overload models by inducing ovarian damage in mice and porcine granulosa cells using pharmacological doses of iron. Nevertheless, this approach has certain limitations, as it cannot fully mimic the chronic and progressive iron overload conditions observed in clinical settings, where excess iron accumulates gradually and continuously in vivo. The dosage, duration, and metabolic dynamics of iron overload in our models are not identical to those in humans with chronic iron metabolism disorders. Therefore, future studies using clinically relevant chronic iron overload models are warranted to further validate the protective effects of spermidine against ovarian dysfunction caused by long-term iron excess.

## 5. Conclusions

Spermidine protected against iron overload-induced ovarian ferroptosis and dysfunction by activating Nrf2/GPX4 antioxidant signaling and AKT-dependent autophagy. Mechanistically, spermidine maintained iron homeostasis through the FHC/TfR1 axis and may regulate glutathione metabolism via the SLC7A11/GSH pathway, which could thereby inhibiting lipid peroxidation, DNA damage, and mitochondrial dysfunction in ovarian granulosa cells. In vivo, spermidine was found to improve estrous cycle regularity, elevate ovarian hormone levels and reduce follicular atresia in iron-overloaded mice. In vitro, spermidine was observed to upregulate the ovarian reserve marker AMH and suppress excessive Nrf2/HO-1 activation. Furthermore, SLC7A11 silencing could abolish the protective effects of spermidine by inducing mitochondrial ROS accumulation and autophagy impairment. Proteomic analysis indicated that MGST3, a mitochondrial protein involved in glutathione metabolism, may act as a putative key downstream target of the spermidine/SLC7A11 axis.

Collectively, our findings uncover a novel mechanism by which spermidine regulates iron homeostasis and ferroptosis through the FHC/SLC7A11 axis, which may offer a potentially promising therapeutic strategy for iron overload-associated female reproductive disorders.

## Figures and Tables

**Table 1:** Primary antibody information for Western blotting and immunofluorescence.

Antibody	Vendor	Cat. No.	Host Species	Application	Dilutions
FHC	Abcam, Cambridge, UK	ab81444	Rabbit	WB	1:1000
SLC7A11	Proteintech, Wuhan, China	26864-1-AP	Rabbit	WB IF	1:1000 1:200
GPX4	Abcam, UK	ab125066	Rabbit	WB IF	1:1000 1:200
ACSL4	Abcam, UK	ab155282	Rabbit	WB	1:1000
Nrf2	Abcam, UK	ab92946	Rabbit	WB	1:5000
p-Nrf2	Abcam, UK	ab76026	Rabbit	WB	1:3000
HO-1	Proteintech, China	10701-1-AP	Rabbit	WB	1:1000
AMH	Abcam, UK	ab229212	Rabbit	WB	1:1000
γH2AX	Abcam, UK	ab11174	Rabbit	WB IF	1:2000 1:500
TfR1	Proteintech, China	10084-2-AP	Rabbit	WB	1:1000
LC3B	Sigma, St. Louis, MO, USA	L7543	Rabbit	WB	1:1000
P62	CST, Danvers, MA, USA	23214S	Rabbit	WB	1:1000
NCOA4	Abmart, Shanghai, China	TD4255	Rabbit	WB	1:1000
AKT	Proteintech, China	10176-2-AP	Rabbit	WB	1:1000
p-Akt	Proteintech, China	28731-1-AP	Rabbit	WB	1:1000
FSHR	Biotechnology, Shanghai, China	AF6930	Rabbit	IF	1:200
β-Actin	TRANS, Beijing, China	HC201-02	Mouse	WB	1:1000
β-Tubulin	Abclonal, Wuhan, China	AC021	Mouse	WB	1:2000

Cat. No.: Catalog Number; WB: Western blot; IF: Immunofluorescence.

## Data Availability

The original data of RNA sequences presented in the study are openly available in NCBI GenBank database with the accession code PZ307866, PZ307867, PZ307868 and PZ307869. Further inquiries can be directed to the corresponding authors.

## Supplementary Materials

The following supporting information can be downloaded at: https://www.mdpi.com/article/10.3390/antiox15050637/s1. Material and methods: Quantitative detection of iron content in feed; Figure S1: Influence of spermidine on FAC-stimulated mitochondrial superoxide (red fluorescent) in granulosa cells; Figure S2: Data quality control; Table S1: Quantitative detection of iron ion content in mouse feed.

## Abbreviations

The following abbreviations are used in this manuscript:

FHC	Ferritin heavy chain
SLC7A11	Solute carrier family 7 member 11
HFe	High-iron group
3SH	3 mm spermidine + high-iron group
ANOVA	Analysis of variance
ACSL4	Acyl-coa synthetase long-chain family member 4
SLC3A2	Solute carrier family 3 member 2
BNC1	Basic nucleoprotein 1
SLC40A1	Solute carrier family 40 (iron-regulated transporter), member 1
FAC	Ferric ammonium citrate
GSH	Glutathione
GSSG	Glutathione disulfide
LPO	Lipid peroxidation products
CQ	Chloroquine
PBS	Phosphate-buffered saline
Rapa	Rapamycin
Fer-1	Ferrostatin-1
Trans	Trans-4-methylcyclohexylamine
Per	Perifosine
PROG	Progesterone
E2	Estradiol
MDA	Malondialdehyde
SOD	Superoxide dismutase
CAT	Catalase
ROS	Reactive oxygen species
DOR	Diminished ovarian reserve
HE staining	Hematoxylin and eosin staining
TEM	Transmission electron microscopy
LIP	Labile iron pool
TFR1	Transferrin receptor
NCOA4	Nuclear receptor coactivator 4
GPX4	Glutathione peroxidase 4
AMH	Anti-Müllerian hormone
ATF4	Activating transcription factor 4
DEPs	Differentially expressed proteins
MGST3	Microsomal glutathione s-transferase 3
