# Supplementary material for: Spermidine Targets Ovarian Granulosa Cells via Activating the FHC/SLC7A11 Axis to Regulate Iron Homeostasis and Ameliorate Iron Overload-Induced Ovarian Dysfunction
Source: Antioxidants (Basel). 2026 May 18;15(5):637. doi: 10.3390/antiox15050637 (PMC13203626; doi:10.3390/antiox15050637)
Supplement: Supplementary file 1 [file antioxidants-15-00637-s001.zip › antioxidants-4273140-supplementary.pdf]

Spermidine targets ovarian granulosa cells via activating the FHC/SLC7A11 axis to regulate iron homeostasis and ameliorate iron overload-induced ovarian dysfunction

Material and methods

Quantitative detection of iron content in feed

Refer to the rodent laboratory animal synthetic feed formula given by the Association of Official Analytical Chemists (AOAC), and adjust the formula appropriately and reasonably, remove the iron in the inorganic salt in the original formula, add different doses of ferrous sulfate to the feed, and prepare feeds with different iron content to improve. Quantitative detection of trace element iron content in feed refers to the national GB/T13885-2017 Determination of calcium, copper, iron, magnesium, manganese, potassium, sodium and zinc content in feed by atomic absorption spectrometry. The operation steps of atomic absorption spectrometry are as follows: weigh 0.2 ~ 0.3 g of feed samples, put them into the ordinary tetrafluoroethylene digestion tank, and supplement with 10 mL of mixed acid (4: 1 nitric acid: perchloric acid) at room temperature overnight hydrolysis, it was placed on an electric heating plate for hydrolysis at 160℃ for 2 h. After cooling, the above 2 mL of perchloric acid and 8 mL of nitric acid were acceded, then heated and digested into colourless. Most of the acid was evaporated. The acid was concentrated to a volume close to 1.0 mL and put to a 50 mL volumetric flask after cooling. According to the lower limit of the estimated value of the content of the measured elements in the feed, 0.5% nitric acid was supplemented to the constant volume and mixed to be measured, and the blank assay was done in the meantime. The national standard sample (standard value: 1000 μg/mL) was obtained from the National Center for Analysis and Detection of Nonferrous Metals and Electronic Materials (iron: GSB 04-1726-2004). Atomic absorption spectrometer (CONTRAA 700; analytik Jena AG, Jena, Germany).

Results

According to the Chinese national trace element detection standard, the error was less than 15%, and the iron content in the feed met the requirements of this experiment (Table S1).

Table S1. Quantitative detection of iron ion content in mouse feed

Group	Repeats	Feeding quality	Concentration	Iron content (mg/kg）	Mean value (mg/kg）
Control（45 mg/kg）	1-1	0.3315	1.5650	47.2097	50.1816
	1-2	0.3268	1.6380	50.1224	
	1-3	0.3572	1.8570	51.9877	
	1-4	0.3235	1.6630	51.4065	
	1-5	0.3611	1.9700	50.1816	
Medium-iron（550 mg/kg）	2-1	0.2975	1.6770	563.6975	584.3361
	2-2	0.3068	1.7390	566.8188	

	2-3	0.3016	1.8110	600.4642	
	2-4	0.3190	1.9370	607.2100	
	2-5	0.3186	1.8590	583.4903	
High-iron（1350 mg/kg）	3-1	0.3019	1.5430	1533.2892	1418.2612
	3-2	0.3083	1.5020	1461.5634	
	3-3	0.3004	1.3440	1342.2104	
	3-4	0.3346	1.5090	1352.9588	
	3-5	0.3115	1.4550	1401.2841	

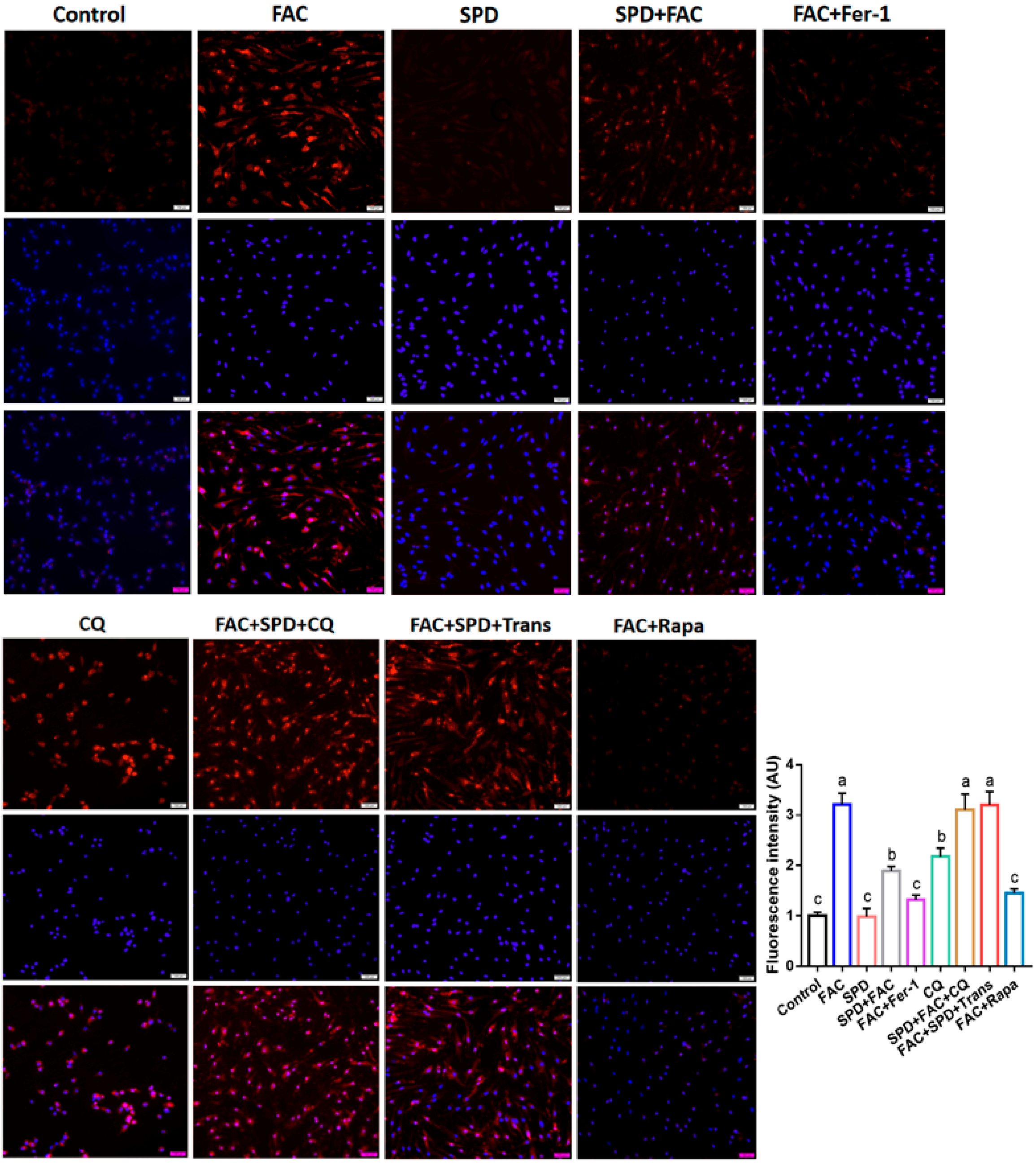

Figure S1. Influence of spermidine on FAC-stimulated mitochondrial superoxide (red fluorescent) in granulosa cells.

The threshold of peptide mass error was ± 10 ppm. Peptide FDR ≤ 0.01 and FDR ≤ 0.01 were used as screening criteria. The peptide scores were mainly distributed between 4000 ~ 8000 (Figure S2A). The correlation coefficient of the specimens in the group was close to 1, indicating a strong correlation (Figure S2B). PCA analysis showed that the specimens in the group were close to each other, indicating high similarity and good repeatability (Figure S2C). The default T test (student t test) combined with the fold change (FC) was used to screen out the significant difference proteins (*P* value = 1.2 or < = 1/1.2).

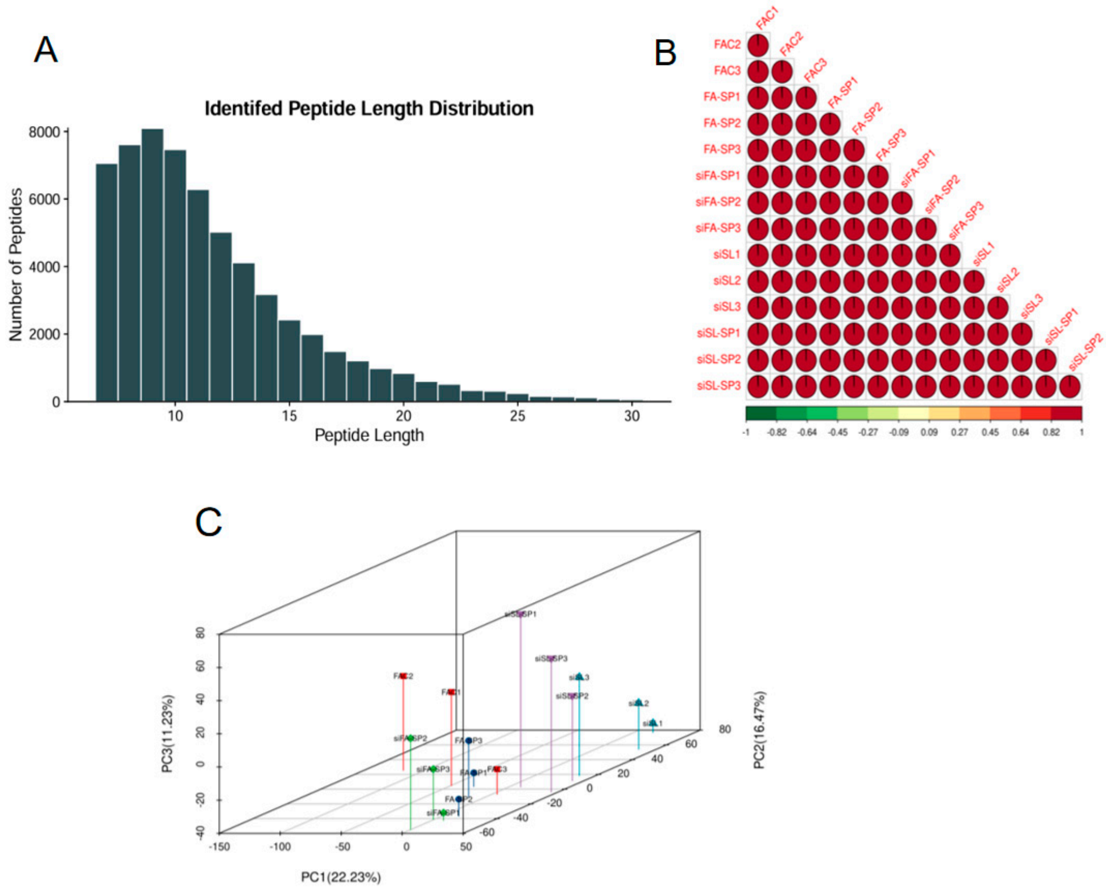

Figure S2. Data quality control. (**A**) Peptide length distribution map. (**B**) Sample correlation diagram (bubble diagram). (**C**) PCA score plot.
